# Gender differences in use of invasive diagnostic and therapeutic procedures for acute ischaemic heart disease in Chinese adults

**DOI:** 10.1136/heartjnl-2021-318988

**Published:** 2021-05-27

**Authors:** Muriel Levy, Yiping Chen, Robert Clarke, Yu Guo, Jun Lv, Canqing Yu, Liming Li, Zhengming Chen, Borislava Mihaylova

**Affiliations:** 1 CTSU, Nuffield Department of Population Health, University of Oxford, Oxford, UK; 2 Health Economics Research Centre, Nuffield Department of Population Health, University of Oxford, Oxford, UK; 3 Medical Research Council Population Health Research Unit (MRC PHRU), Nuffield Department of Population Health, University of Oxford, Oxford, UK; 4 Department of Epidemiology, Peking University Health Science Centre, Beijing, China; 5 Department of Epidemiology and Biostatistics, Chinese Academy of Medical Sciences, Beijing, China; 6 Institute of Population Health Sciences, Queen Mary University of London, London, UK

**Keywords:** gender, coronary angiography, percutaneous coronary intervention, epidemiology, healthcare economics and organisations

## Abstract

**Objective:**

To investigate gender differences in the use of diagnostic and therapeutic procedures for acute ischaemic heart disease (IHD) in Chinese adults and assess whether socioeconomic or health system factors contribute to such differences.

**Methods:**

In 2004–2008, the China Kadoorie Biobank recruited 512 726 adults from 10 diverse areas in China. Data for 38 928 first hospitalisations with IHD (2911 acute myocardial infarction (AMI), 9817 angina and 26 200 other IHD) were obtained by electronic linkage to health insurance records until 31 December 2016. Multivariate Poisson regression models were used to estimate women-to-men rate ratios (RRs) of having cardiac enzyme tests, coronary angiography and coronary revascularisation.

**Results:**

Among the 38 928 individuals (61% women) with IHD admissions, women were less likely to have AMI (5% vs 12%), but more likely to have angina (26% vs 24%) or other IHD (69% vs 64%). For admissions with AMI, there were no differences in the use of cardiac enzymes between women and men (RR=1.00; 95% CI, 0.97 to 1.03), but women had lower use of coronary angiography (0.80, 0.68 to 0.93) and coronary revascularisation (0.85, 0.74 to 0.99). For angina, the corresponding RRs were: 0.97 (0.94 to 1.00), 0.66 (0.59 to 0.74) and 0.56 (0.47 to 0.67), respectively; while for other IHD, they were 0.97 (0.94 to 1.00), 0.87 (0.76 to 0.99) and 0.61 (0.51 to 0.73), respectively. Adjusting for socioeconomic and health system factors did not significantly alter the women-to-men RRs.

**Conclusions:**

Among Chinese adults hospitalised with acute IHD, women were less likely than men to have coronary angiography and revascularisation, but socioeconomic and health system factors did not contribute to these differences.

## Introduction

Ischaemic heart disease (IHD) is a leading cause of premature death in China, accounting for >1.6 million deaths in 2016.^1^ Previous studies have reported a lower incidence of acute coronary syndrome (ACS) in women compared with men, but women have a higher case fatality rate following ACS than men.^2 3^ In addition to differences in disease severity, differences in the use of invasive diagnostic and therapeutic procedures for acute IHD could also contribute to differences in the prognosis of acute IHD between men and women.^2^ Women experience longer delays in access to hospital care and are less likely than men to have invasive diagnostic procedures,^3 4^ but the reasons for such differences in the clinical management of acute IHD remain unexplained.

In recent decades, access to diagnostic and therapeutic procedures for acute IHD have improved substantially in low-income and middle-income countries. In China, the healthcare system has undergone major reforms, with substantial investment in hospitals, improvement in education of health professionals and medical equipment, and better access to hospital care for lower socioeconomic groups.^5 6^ Nevertheless, out-of-pocket (OOP) expenses as percentage of total healthcare expenditure have remained high in China,^7^ and women remain less likely to use inpatient care and more likely to defer use of healthcare until urgent for economic reasons.^8–10^

Previous studies examined differences in the use of invasive diagnostic tests and procedures for ACS between men and women in China, but were constrained by restriction to tertiary hospitals, lack of longitudinal data, and failure to investigate the effects of socioeconomic or health system factors.^11 12^ The aims of the present study were: (1) to examine differences in the use of invasive diagnostic and therapeutic procedures for acute myocardial infarction (AMI), angina and other IHD in Chinese men and women; and (2) to investigate the extent to which socioeconomic or health system factors could explain such differences.

## Methods

### Study population

The China Kadoorie Biobank (CKB) is a prospective study of 512 726 adults, aged 30–79 years at entry, who were recruited from five urban and five rural areas in China between 25 June 2004 and 15 July 2008.^13 14^All participants completed an interviewer-administered questionnaire, providing detailed information on demographic and socioeconomic characteristics, medical history, and lifestyle factors. Physical measurements were also recorded.

### Socioeconomic and health system factors

Socioeconomic factors, collected at entry into the study (2004–2008), included marital status, household size, highest level of education attained and annual household income. Health insurance (HI) types at admission were categorised into Urban Employee Basic Medical Insurance (UEBMI), Urban Resident Basic Medical Insurance (URBMI) or New Rural Cooperative Medical Scheme (NRCMS), and uninsured or others. Hospitals were classified as tier 3 (≥500 beds), tier 2 (100–500 beds), tier 1 (<100 beds) hospitals, and hospitals with unspecified or missing tier (online supplemental appendix p4–5).

10.1136/heartjnl-2021-318988.supp1 Supplementary data

### Hospital admissions for IHD

Data on hospital admissions for acute IHD were obtained by linkage via the unique national identification number to electronic hospital records from the nationwide health insurance system and to regional IHD registers. Diagnoses of acute IHD, recorded during hospital admissions, were reviewed, integrated centrally and standardised using the International Classification of Diseases 10th revision (ICD-10) codes. Hospital admissions for AMI, angina pectoris and other IHD were identified using ICD-10 codes I21, I20 and I22–I25, respectively. Admissions for other IHD involved mostly cases with non-specific atherosclerotic heart diseases (I25.1, 92%). The present analyses were restricted to participants’ first hospitalisations for acute IHD recorded during the study in any hospital between 25 June 2004 and 31 December 2016.

### Outcome measures

Primary outcomes compared use of non-invasive cardiac enzyme tests with invasive diagnostic procedures (coronary angiography during the first acute IHD admission after entry into the study) and invasive therapeutic procedures (coronary revascularisation within 3 months of admission for acute IHD). Cardiac enzyme tests included creatine kinase-MB or troponins, and coronary revascularisation procedures chiefly involved percutaneous coronary intervention (PCI) (few participants had coronary artery bypass graft operations). The secondary outcomes included ECG, echocardiogram, Holter monitor tests and coronary CT angiography (CCTA).

Data on coronary revascularisation procedures were obtained from HI records, supplemented by retrieved medical records. Data on cardiac enzyme tests, coronary angiography and secondary outcomes were obtained from retrieved medical records for 24 408 IHD cases that underwent clinical adjudication by certified cardiologists in China (online supplemental appendix p5).

### Patient and public involvement

Prior to recruitment in CKB, local community leaders in China were consulted. The study findings are reported in peer-reviewed publications and any relevant public health messages disseminated using local press, television and internet.

### Statistical analysis

All analyses were performed separately for AMI, angina and other IHD. Missing values for length of hospital stay (6% of IHD cases) were imputed using multiple imputation (online supplemental appendix p6). The women-to-men (ie, men as the reference group) rate ratios (RRs) of having a diagnostic test or procedure were estimated using Poisson regression with robust variance estimation to minimise overestimation of variance for binary data.^15^ Goodness-of-fit statistics confirmed model adequacy. Age-adjusted and region-adjusted rates of having a diagnostic test or procedure per 100 admissions were estimated in men and women, by age group (<55 years, 55–65 years and ≥65 years old). Models were sequentially adjusted for demographic, lifestyle and morbidity risk factors, followed by HI type, socioeconomic factors and hospital tier. For tests and procedures with statistically significant women-to-men RRs, effect modification by socioeconomic and health system factors was assessed by adding interaction terms to the Poisson regression models. Heterogeneity or trends in women-to-men RRs of having diagnostic tests or procedures were assessed using Χ^2^ tests by categories of selected factors.

Sensitivity analyses included analyses of use of tests and procedures for ST-segment elevation MI (STEMI) and non-STEMI (NSTEMI) in a subsample of AMI admissions with retrieved medical records (60%); first-ever AMI, angina and other IHD (ie, excluding participants with previous cardiovascular disease (CVD)); and stratified by region. Further analyses estimated women-to-men RRs by categories of established IHD risk factors, and 28-day case fatality rates. All analyses were performed using Stata V.15 or R V.3.6.0.

## Results

Between 2004 and 2016, 38 928 participants (61% women) were admitted to hospital with a first episode of IHD, including 2911 (8%) with AMI, 9817 with angina (25%) and 26 200 (67%) with other IHD (table 1). Compared with men, women were less likely to be admitted with AMI (5% vs 12% of respective admissions), but more likely to be admitted with angina (26% vs 24%) and other IHD (69% vs 64%). Women admitted with AMI were older than men at baseline (mean age 61.7 vs 58.7 years), and at admission to hospital (67.8 vs 64.7: table 1). Compared with men, women with AMI had a higher prevalence of diabetes, overweight or obesity, self-rated poor health status, mental illness, and higher mean systolic blood pressure and lower physical activity levels at baseline and a lower prevalence of prior stroke or transient ischaemic attack, or being a current smoker or drinker. Women were less likely to be married (77.6% vs 94.5%), have completed high school or above (14.1% vs 30.1%), have an annual household income above ¥20 000 (32.6% vs 47.2%), live in urban areas (60.9% vs 64.4%) and be enrolled in the UEBMI scheme (51.0% vs 62.8%).

**Table 1:** Selected characteristics of men and women with hospital admission for acute MI, angina and other IHD during 2004–2016

	Acute MI			Angina			Other IHD		
	Men (n=1799)	Women (n=1112)	P value	Men (n=3626)	Women (n=6191)	P value	Men (n=9792)	Women (n=16 408)	P value
**(A) Characteristics at baseline**									
Age (years), mean (SD)	58.7 (10.1)	61.7 (9.2)	**	58.0 (10.2)	58.0 (9.5)		59.8 (10.1)	58.7 (9.6)	**
**Self-reported medical history, %**									
Diabetes†	14.8	25.1	**	12.6	13.2		11.6	13.1	**
Hypertension†	55.0	58.4		51.4	44.5	**	53.4	46.8	**
Stroke or TIA	6.7	3.7	**	5.1	3.6	**	6.0	3.8	**
IHD	11.4	13.3		16.4	19.7	**	14.5	14.6	
CKD	1.6	1.9		2.7	3.4		2.3	2.9	*
Poor health status	12.0	17.8	**	12.1	15.3	**	15.0	20.2	**
Mental illness‡	9.6	12.6	**	10.0	13.4	**	10.6	13.5	**
**Physical measurements**									
Overweight or obese (>25 kg/m²), %	43.5	47.8	*	47.3	49.2		40.2	46.9	**
SBP (mm Hg), mean (SD)	141.8 (22.4)	144.4 (24.6)	**	138.6 (20.9)	134.9 (23.3)	**	139.9 (21.8)	137.1 (23.8)	**
**Lifestyle characteristics**									
Current smoker, %	61.6	7.3	**	53.9	3.5	**	52.8	5.0	**
Regular alcohol drinker, %	40.2	3.3	**	46.5	5.1	**	42.9	4.8	**
Physical activity (MET—hour/day), mean (SD)	15.5 (13.4)	13.3 (9.0)	**	14.9 (11.8)	13.6 (8.6)	**	15.1 (12.9)	14.7 (9.6)	**
**Socioeconomic characteristics**									
Currently married, %	94.5	77.6	**	93.6	83.5	**	91.2	82.1	**
Household size, mean (SD)	3.5 (1.6)	3.5 (1.8)		3.4 (1.5)	3.2 (1.5)	**	3.5 (1.6)	3.4 (1.6)	**
High school or above, %	30.1	14.1	**	38.7	30.0	**	30.1	20.3	**
Annual household income > ¥20 000, %	47.2	32.6	**	52.3	46.3	**	43.8	36.3	**
Rural residents, %	35.6	39.1		31.4	23.5	**	48.2	46.4	**
**(B) Characteristics at hospital admission**									
Age (years), mean (SD)	64.7 (10.0)	67.8 (9.1)	**	64.0 (10.1)	64.1 (9.3)		66.2 (10.2)	65.0 (9.8)	**
**Health insurance type§, %**			**			*			**
NRCMS or URBMI	36.0	48.1		29.8	28.2		44.7	48.1	
UEBMI	62.8	51.0		69.4	71.4		54.7	51.7	
Other or uninsured	1.2	0.9		0.7	0.5		0.6	0.3	
**Hospital tier, %**			**						**
Tier 1 or missing/unspecified	9.1	12.1		16.0	14.7		31.3	36.2	
Tier 2	15.3	17.4		18.7	18.8		19.3	18.8	
Tier 3	75.7	70.5		65.3	66.6		49.4	45.1	
**Length of stay (days)¶, mean** (**SD**)	10.9 (6.7)	10.8 (6.9)		10.0 (5.9)	10.3 (5.4)		9.9 (11.1)	9.8 (10.2)	

*P value of <0.05, **p value of <0.01.

†Self-reported and screen detected.

‡Mental illness was defined as having at least one symptom of depression or anxiety in the past 12 months.

§Data on health insurance (HI) types for each participant was identified annually in 2012–2016, but was unavailable for the years prior to 2012. Missing data on HI type in 2004–2011 were imputed based on the insurance scheme in which participants were enrolled in 2012.

¶Missing length of stay for 6% of all IHD admissions imputed using multiple imputation.

CKD, chronic kidney disease; IHD, ischaemic heart disease; MET, metabolic equivalents of task; MI, myocardial infarction; NRCMS, New Rural Cooperative Medical Scheme; SBP, systolic blood pressure; TIA, transient ischaemic attack; UEBMI, Urban Employee Basic Medical Insurance; URBMI, Urban Resident Basic Medical Insurance.

Differences in baseline characteristics between men and women admitted with angina or other IHD were similar, although less extreme than those for AMI (table 1). Women with AMI or other IHD were more likely than men to be admitted to smaller (tier 1) rather than larger (tier 3) hospitals (table 1). Duration of hospital stay did not differ by gender for AMI, angina and other IHD (mean: 10–11 days). Overall, the baseline characteristics of individuals with retrieved medical records (n=24 408) were comparable with those of all individuals with AMI, angina and other IHD (online supplemental table 1).

### Use of diagnostic tests and procedures

**Figure 1:**
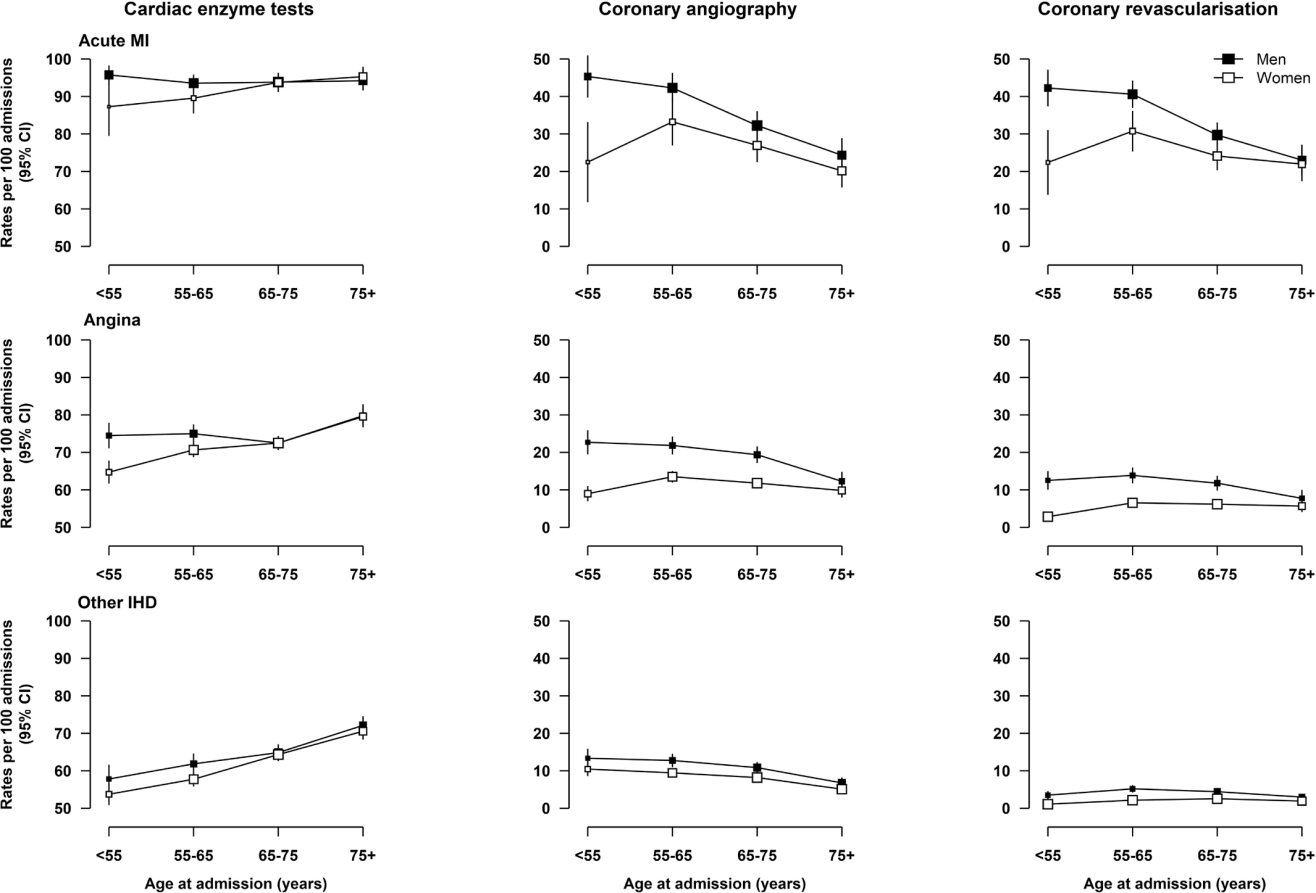
Age-adjusted and region-adjusted rates of having cardiac enzyme tests, coronary angiography and coronary revascularisation per 100 admissions for acute MI, angina and other IHD in men and women, by age group. Poisson models with adjustments for age in years and region were used. Rates were standardised for the overall CKB participant population with acute MI and other IHD, respectively. Coronary revascularisations within 3 months post-admission were included. CKB, China Kadoorie Biobank; IHD, ischaemic heart disease; MI, myocardial infarction.

Among AMI admissions, almost 95% had cardiac enzyme tests, with no material differences between men and women (figure 1). In contrast, after adjusting for age and region, among AMI cases, women had lower rates than men of coronary angiography (28% vs 35%) and coronary revascularisation (26% vs 33%). Likewise among cases with angina and other IHD, women had comparable rates with men of use of cardiac enzyme tests (72% vs 76% and 62% vs 65%, respectively), but much lower rates of coronary angiography (12% vs 20% and 8% vs 11%) and coronary revascularisation (6% vs 12% and 2% vs 4%). These differences in the use of invasive coronary investigations attenuated with increasing age (figure 1).

**Figure 2:**
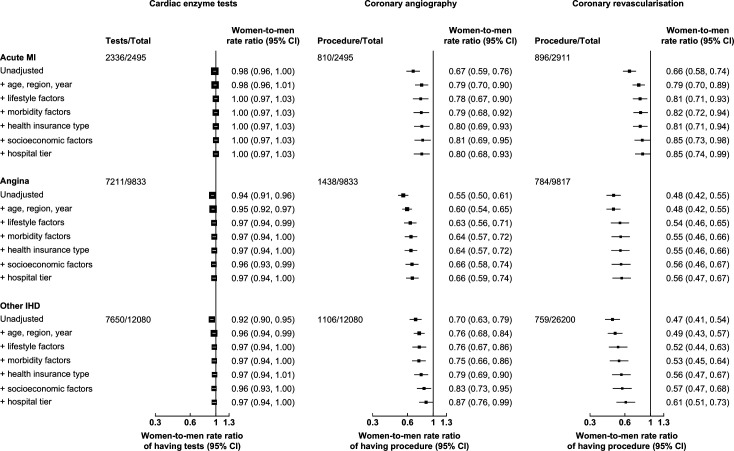
Adjusted women-to-men rate ratios of having cardiac enzyme tests, coronary angiography and coronary revascularisation for acute MI, angina and other IHD, after sequential adjustment for confounding factors. Lifestyle factors included smoking, alcohol consumption, body mass index and physical activity. Morbidity factors included systolic blood pressure, self-rated health status, mental illness, self-reported doctor-diagnosed diseases at entry into CKB, with updated histories of IHD, cerebrovascular disease, malignant neoplasms, respiratory diseases, infectious and parasitic diseases, diabetes mellitus and chronic kidney disease. Socioeconomic factors included marital status, household size, education and income. The total number for analyses of cardiac enzyme tests and coronary angiography included first admissions for participants with retrieved medical records. The total number for analyses of coronary revascularisation included all first IHD admissions for participants with and without retrieved medical records. Coronary revascularisations within 3 months post-admission were included. The area of each square is inversely proportional to the variance. CKB, China Kadoorie Biobank; IHD, ischaemic heart disease; MI, myocardial infarction.

Following adjustment for all relevant factors, the women-to-men RRs of having cardiac enzyme tests, coronary angiography and coronary revascularisation increased marginally for AMI, angina and other IHD. The attenuation in differences was mainly due to adjustment for age and region. Further adjustment for HI type, socioeconomic factors and hospital tier did not materially alter the women-to-men RRs of having cardiac enzyme tests, coronary angiography and coronary revascularisation. The fully adjusted RRs of having diagnostic tests and procedures for AMI were: 1.00 (95% CI 0.97 to 1.03) for cardiac enzyme tests, 0.80 (0.68 to 0.93) for coronary angiography and 0.85 (0.74 to 0.99) for coronary revascularisation. The corresponding RRs for angina were 0.97 (0.94 to 1.00), 0.66 (0.59 to 0.74) and 0.56 (0.47 to 0.67); and those for other IHD were 0.97 (0.94 to 1.00), 0.87 (0.76 to 0.99) and 0.61 (0.51 to 0.73), respectively (figure 2).

**Figure 3:**
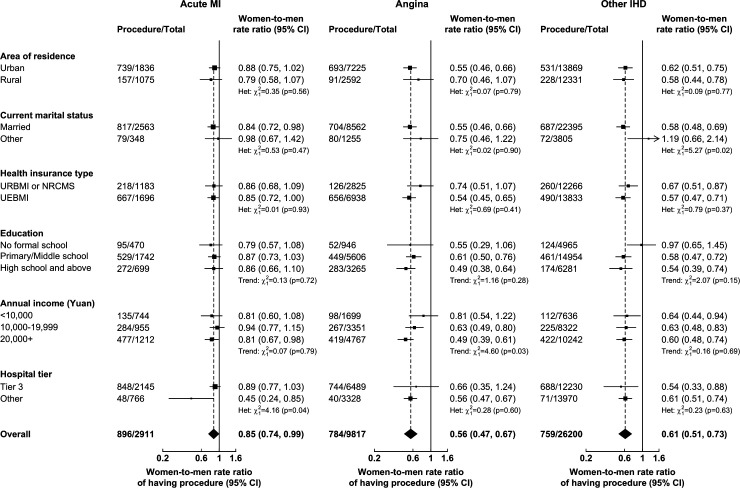
Adjusted women-to-men rate ratios of having coronary revascularisation for acute MI, angina and other IHD, by socioeconomic and health system factors. In analyses by health insurance type, uninsured participants were excluded due to small number of cases. Models included adjustments for demographic factors, lifestyle factors, morbidity factors, health insurance type (except by area of residence), socioeconomic factors and hospital tier, as appropriate. Coronary revascularisations within 3 months post-admission were included. The area of each square is inversely proportional to the variance. IHD, ischaemic heart disease; MI, myocardial infarction; NRCMS, New Rural Cooperative Medical Scheme; UEBMI, Urban Employee Basic Medical Insurance; URBMI, Urban Resident Basic Medical Insurance.

Among cases with AMI, the fully adjusted women-to-men RRs of having coronary procedures did not vary significantly by area of residence, marital status, HI type, education and income (figure 3, online supplemental figure 1), but were higher for individuals admitted to tier 3 hospitals (coronary angiography: heterogeneity p=0.03, revascularisation: p=0.04). Among cases with angina and other IHD, there was little evidence of heterogeneity in the adjusted women-to-men RRs of having coronary procedures between different participant categories with the exception of income (both procedures for angina) and marital status (revascularisation for other IHD) (figure 3, online supplemental figure 1). Across categories of IHD admissions, the rates of having cardiac enzyme tests, coronary angiography and revascularisation were higher in urban than in rural areas, and in tier 3 than lower tier hospitals. The rates of having cardiac enzyme tests and coronary angiography were also higher for individuals enrolled in UEBMI than in those enrolled in URBMI or NRCMS (online supplemental tables 2–4). Rates of coronary procedures following AMI increased with levels of education in both men and women (online supplemental table 2).

In sensitivity analyses, using a subsample of AMI admissions with available information on subtypes of AMI, the proportions with STEMI were 52% in women and 68% in men. The women-to-men RRs of having coronary procedures were similar for STEMI and NSTEMI subtypes (online supplemental figure 2). The minimal effect of adjusting for socioeconomic and health system factors on the women-to-men RRs of having cardiac tests and procedures persisted for both STEMI and NSTEMI (online supplemental figure 2), after excluding individuals with prior CVD (online supplemental figure 3) and after stratification by region (online supplemental figure 4).

In further analyses, the adjusted women-to-men RRs of having coronary angiography and revascularisation for AMI did not vary by levels of established risk factors (online supplemental figure 5). In cases with angina, the RRs of having coronary procedures varied by age, hypertension and diabetes, while for participants with other IHD, the RRs of having revascularisation varied by age and prior CVD (online supplemental figures 6 and 7). For all individuals with acute IHD, the rates of having cardiac tests and procedures increased over time (online supplemental tables 2–4). The rates of having ECG, echocardiogram, Holter monitor tests and CCTA were comparable between men and women (online supplemental table 5). Among AMI admissions, the adjusted 28-day case fatality rates did not differ significantly (women-to-men RR: 0.84 (0.63 to 1.11)). A small proportion of admissions for angina and other IHD resulted in death within 28 days, although the case fatality rates for other IHD were higher in men than women (online supplemental table 6).

## Discussion

This study of Chinese adults admitted into hospital with acute IHD demonstrated that the use of invasive coronary artery procedures was substantially lower in women compared with men. Moreover, these differences were greater in younger individuals, but were similar in urban and rural areas. While women had lower socioeconomic status than men and were more likely to be admitted to lower tier hospitals, differences in socioeconomic and health system factors explained only a small fraction of the gender differences in the use of invasive diagnostic or therapeutic procedures.

Contemporary international and Chinese treatment guidelines for ACS have similar recommendations for the use of invasive diagnostic and therapeutic procedures in men and women.^3 16–18^ However, previous studies conducted in China also reported lower use of coronary angiography and revascularisation in women compared with men.^11 12^ A nationwide registry study of STEMI cases recruited from mostly tertiary hospitals in China in 2014–2018 reported that women had lower rates of primary PCI than men (44% vs 51%).^11^ Likewise, the China PEACE-Retrospective Study, involving 162 hospitals in 2001–2011, reported that among patients with STEMI, women had lower rates of coronary angiography (19% vs 29%) and PCI (9% vs 14%).^12^ Previous studies had suggested that socioeconomic inequalities might account for some of the differences in access to healthcare services, especially more expensive ones.^19 20^ The present study highlighted differences in the management of acute IHD between women and men in China and investigated the contribution of socioeconomic and health system factors.

Consistent with previous findings, women in CKB had lower socioeconomic status compared with men, evidenced by lower educational attainment and household income.^8 9 20^ They were more likely to be enrolled in HI types with less generous coverage and seek care in lower rank hospitals with more limited expertise and capacity for performing complex procedures. These gender differences in socioeconomic status are likely to be exacerbated by substantial OOP contributions to hospitalisation costs in China. The OOP payments for individuals with AMI requiring revascularisation procedures have been linked with catastrophic health expenditure, especially for individuals from lower socioeconomic groups.^21^ While the magnitude of gender differences in the use of coronary angiography and revascularisation were similar for AMI, they were greater for coronary revascularisation than for coronary angiography among those with angina and other IHD. The differences suggest that financial affordability may influence the use of expensive procedures, particularly in less severe acute IHD cases. Nevertheless, the adjustment for socioeconomic and health system factors accounted for only a small proportion of the gender differences in the use of coronary procedures. It is likely that factors related to disease severity or differences in clinical presentation are more likely to explain the observed gender differences in the use of invasive coronary procedures.

Differences in the use of invasive procedures may reflect heterogeneity in the clinical presentation and pathophysiology of IHD between men and women, as suggested by previous studies.^3 22^ Women with ACS are more likely to present with atypical symptoms that, particularly at younger ages, are stress related, which makes the diagnostic evaluation and subsequent management more challenging. In addition, women have a higher prevalence of non-occlusive coronary artery disease, microvascular dysfunction and spontaneous coronary artery dissection (SCAD) compared with men. Although the present study assessed gender differences separately in cases with AMI, angina and other IHD, we could only differentiate between STEMI and NSTEMI in a subsample of all AMI admissions. While the benefits of an early invasive strategy for STEMI in both men and women are well established, those for NSTEMI are more uncertain, but recent evidence advocates comparable use of coronary procedures in men and women with elevated biomarkers.^23 24^ Previous studies have reported that most AMI cases in both Chinese men and women were STEMI.^25 26^ Although there were no differences in the use of cardiac enzyme tests, the lack of sex-specific cardiac biomarker thresholds may have underestimated the severity of ACS in women, resulting in greater risk of diagnosis misclassification.^27^

The differences in the use of coronary artery procedures among women with AMI compared with men may reflect physician bias or concerns about the safety of such procedures in women.^4^ Women have higher risks than men of bleeding and other vascular complications following PCI, which may prompt a greater reluctance to use coronary revascularisation.^28 29^ Alternatively, unconscious bias or beliefs that AMI or IHD preferentially affect men could lead physicians to underestimate the severity of IHD in women and contribute to a lower use of certain invasive procedures.^2 30^ Such biases may be even more extreme in younger patients due to lower incidence of IHD in younger women.^12^

Despite the large population studied, the diversity of regions and hospitals examined, and the extensive data collected, the present study had several limitations. First, the CKB study was not nationally representative, as participants were recruited from 10 diverse areas rather than being representative of the overall Chinese population. Second, the study could not differentiate AMI subtypes in all cases or exclude alternate diagnoses such as Takotsubo cardiomyopathy or SCAD. Third, although medical records were not retrieved for all participants, results were unlikely to be affected by bias, as differences were similar in individuals with and without retrieved medical records. Fourth, the present study lacked information on the clinical indication for revascularisation procedures, such as echocardiographic or angiographic variables or haemodynamic parameters, delay in admission from onset of acute IHD symptoms, timing of procedure following admission or reasons for not having more invasive procedures. Although interhospital transfers could not be reliably identified in the CKB, the probability of 30-day readmission for individuals with acute IHD admitted to tier 1 hospitals (4%) did not vary by gender. Thus, the present study could not fully exclude residual confounding. Given the rapidly evolving field, future research should investigate current use of invasive procedures for acute IHD in men and women.

Overall, this large prospective study demonstrated that Chinese women were less likely than men to have coronary angiography and coronary revascularisation procedures following hospital admission for AMI, angina and other IHD, but socioeconomic and health system factors did not contribute to these differences. Further systematic monitoring of clinical care in men and women is required as the reasons for differences in the use of invasive coronary procedures remain unexplained.

**Key messages**

**What is already known on this subject?**

- Previous studies have highlighted gender differences in the use of invasive diagnostic and therapeutic procedures for acute ischaemic heart disease (IHD) in high-income countries.
- However, the reasons for such gender differences in clinical care of acute IHD remain largely unexplained.

**What might this study add?**

- Among Chinese adults admitted to hospital with acute IHD, the use of coronary angiography and revascularisation was also substantially lower in women than in men.
- While Chinese women had lower socioeconomic status than men and were more likely to be admitted to lower tier hospitals, differences in socioeconomic and health system factors did not contribute to the differences in the use of such procedures between men and women.

**How might this impact on clinical practice?**

- The lower use of invasive coronary procedures for acute IHD in women than men may reflect sex differences in the clinical presentation or pathophysiology of IHD, or possibly physician bias or concerns about the safety of such procedures in women.

## Data Availability

Selected data on questionnaire and outcome measures are made available to bona fide scientists on reasonable request.
