# Correction: *Gender differences in use of invasive diagnostic and therapeutic procedures for acute ischaemic heart disease in Chinese adults*

**DOI:** 10.1136/heartjnl-2021-318988corr1

**Published:** 2022-07-26

Levy M, Chen Y, Clarke R, *et al*. Gender differences in use of invasive diagnostic and therapeutic procedures for acute ischaemic heart disease in Chinese adults. *Heart* 2022;**108:**292–299.

This article has been corrected since it was first published. In Figure 3, the women-to-men rate ratios by hospital tier for Angina and Other IHD were inverted between ‘Tier 3’ and ‘Other’. This has now been corrected and the new figure is below:


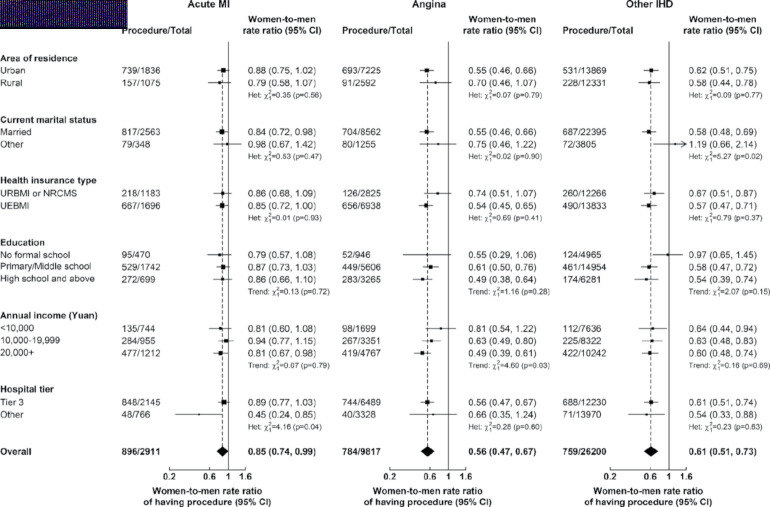


Additionally, the supplementary appendix has been resupplied to make the following corrections to eFigure 1 and eFigure 7:

**A**: Acute MI: women-to-men rate ratios for other (non-married) should be 1.02 (0.68, 1.53) instead of 0.77 (0.66, 0.91)

**C**: Other IHD: women-to-men rate ratios for other hospital tiers should be 0.83 (0.55, 1.24) instead of 0.89 (0.73, 1.09).

