# Supplementary material for: Gender differences in use of invasive diagnostic and therapeutic procedures for acute ischaemic heart disease in Chinese adults
Source: Heart. 2021 May 27;108(4):292–9. doi: 10.1136/heartjnl-2021-318988 (PMC8819660; doi:10.1136/heartjnl-2021-318988)
Supplement: Supplementary data [file heartjnl-2021-318988supp001.pdf]

Supplementary Appendix

Supplement to: Levy M, Chen Y, Clarke R, et al. Gender differences in use of invasive diagnostic and therapeutic procedures for acute ischaemic heart disease in Chinese adults

Levy M, *et al. Heart* 2022; 108:292–299. doi: 10.1136/heartjnl-2021-318988

Table of Contents

Page	
2	Table of contents
3	Members of the China Kadoorie Biobank Collaborative Group
5	Supplementary Methods
9	eTable 1: Selected characteristics of men and women with retrieved medical records for hospital admissions for acute MI, angina, and other IHD, in 2004-2016
10	eTable 2: Adjusted rates (95% CI) of having cardiac enzyme test, coronary angiography and coronary revascularisation for acute MI per 100 admissions for men and women, by socioeconomic and health system factors
11	eTable 3: Adjusted rates (95% CI) of having cardiac enzyme test, coronary angiography and coronary revascularisation for angina per 100 admissions for men and women, by socioeconomic and health system factors
12	eTable 4: Adjusted rates (95% CI) of having cardiac enzyme test, coronary angiography and coronary revascularisation for other IHD per 100 admissions for men and women, by socioeconomic and health system factors
13	eTable 5: Adjusted rates (95% CI) of having other diagnostic tests for acute MI, angina, and other IHD per 100 admissions for men and women
13	eTable 6: Adjusted women-to-men rate ratios and rates of 28-days case fatality for acute MI and other IHD for men and women
14	eFigure 1: Adjusted women-to-men rate ratios of having coronary angiography for acute MI, angina, and other IHD, by socioeconomic and health system factors
15	eFigure 2: Adjusted women-to-men rate ratios of having cardiac enzyme tests, coronary angiography and coronary revascularisation for STEMI and NSTEMI, after sequential adjustment for confounding factors
16	eFigure 3: Adjusted women-to-men rate ratios of having cardiac enzyme test, coronary angiography and coronary revascularisation for first-ever acute MI, angina and other IHD, by level of adjustment
17	eFigure 4: Adjusted women-to-men rate ratios of having cardiac enzyme test, coronary angiography and coronary revascularisation for acute MI, angina and other IHD by level of adjustment, stratified by region
18	eFigure 5: Adjusted women-to-men rate ratios of having cardiac enzyme test, coronary angiography and coronary revascularisation for acute MI, by selected risk factors

Levy M, *et al. Heart* 2022; 108:292–299. doi: 10.1136/heartjnl-2021-318988

BMJ Publishing Group Limited (BMJ) disclaims all liability and responsibility arising from any reliance placed on this supplemental material which has been supplied by the author(s)

19 eFigure 6: Adjusted women-to-men rate ratios having cardiac enzyme test, coronary angiography and coronary revascularisation for angina, by selected risk factors

20 eFigure 7: Adjusted women-to-men rate ratios having cardiac enzyme test, coronary angiography and coronary revascularisation for other IHD, by selected risk factors

Members of the China Kadoorie Biobank Collaborative Group

International Steering Committee: Junshi Chen, Zhengming Chen (PI), Robert Clarke, Rory Collins, Yu Guo, Liming Li (PI), Jun Lv, Richard Peto, and Robin Walters.

International Co-ordinating Centre, Oxford: Daniel Avery, Derrick Bennett, Ruth Boxall, Fiona Bragg, Sushila Burgess, Kahung Chan, Yumei Chang, Yiping Chen, Zhengming Chen, Robert Clarke, Huaidong Du, Zammy Fairhurst-Hunter, Wei Gan, Simon Gilbert, Alex Hacker, Parisa Hariri, Michael Holmes, Andri Iona, Becky Im, Maria Kakkoura, Christiana Kartsonaki, Rene Kerosi, Garry Lancaster, Kuang Lin, John McDonnell, Iona Millwood, Qunhua Nie, Alfred Pozaricki, Paul Ryder, Sam Sansome, Dan Schmidt, Rajani Sohoni, Becky Stevens, Iain Turnbull, Robin Walters, Lin Wang, Neil Wright, Ling Yang, Xiaoming Yang and Pang Yao.

National Co-ordinating Centre, Beijing: Zheng Bian, Yu Guo, Xiao Han, Can Hou, Chao Liu, Jun Lv, Pei Pei,Canqing Yu and Chun Li.

Regional Co-ordinating Centres: Qingdao **Qingdao** CDC: Zengchang Pang, Ruqin Gao, Shanpeng Li, Shaojie Wang, Yongmei Liu, Ranran Du, Liang Cheng, Xiaocao Tian, Hua Zhang, Yaoming Zhai, Feng Ning, Xiaohui Sun, Feifei Li. Licang CDC: Silu Lv, Junzheng Wang, Wei Hou. **Heilongjiang** Provincial CDC: Mingyuan Zou, Shichun Yan, Xue Zhou. Nangang CDC: Bo Yu, Yanjie Li, Qinai Xu, Quan Kang, Ziyan Guo. **Hainan** Provincial CDC: Ximin Hu, Jinyan Chen, Xiaohuan Wang. Meilan CDC: Min Weng, Zhendong Guo, Shukuan Wu, Yilei Li, Huimei Li. **Jiangsu** Provincial CDC: Ming Wu, Yonglin Zhou, Jinyi Zhou, Ran Tao, Jie Yang, Jian Su. Suzhou CDC: Fang Liu, Jun Zhang, Yihe Hu, Yan Lu, Liangcai Ma, Aiyu Tang, Yujie Hua, Jianrong Jin, Jingchao Liu. **Guangxi** Provincial CDC: Zhenzhu Tang, Naying Chen, Duo Liu. Liuzhou CDC: Mingqiang Li, Jinhuai Meng, Rong Pan, Qilian Jiang, Jian Lan, Yun Liu, Liuping Wei, Liyuan Zhou, Ningyu Chen, Ping Wang, Fanwen Meng, Yulu Qin, Sisi Wang. **Sichuan** Provincial CDC: Xianping Wu, Ningmei Zhang, Xiaofang Chen, Weiwei Zhou. Pengzhou CDC: Guojin Luo, Jianguo Li, Xiaofang Chen, Xunfu Zhong, Jiaqiu Liu, Qiang Sun. **Gansu** Provincial CDC: Pengfei Ge, Xiaolan Ren, Caixia Dong. Maiji CDC: Hui Zhang, Enke Mao, Zhongxiao Li, Tao Wang, Xi Zhang. **Henan** Provincial CDC: Ding Zhang, Gang Zhou, Shixian Feng, Liang Chang, Lei Fan. Huixian CDC: Yulian Gao, Tianyou He, Huarong Sun, Pan He, Chen Hu, Xukui Zhang. **Zhejiang** Provincial CDC: Min Yu, Ruying Hu, Hao Wang, Weiwei Gong, Meng Wang. Tongxiang CDC: Chunmei Wang, Xiaoyi Zhang, Kaixu Xie, Lingli Chen, Dongxia Pan, Qijun Gu. **Hunan** Provincial CDC: Yuelong Huang, Biyun Chen, Li Yin, Huilin Liu, Zhongxi Fu, Qiaohua Xu. Liuyang CDC: Xin Xu, Hao Zhang, Huajun Long, Libo Zhang.

IHD Adjudication Committee: Qiling Chen, Xiaoli Cheng, Xiaomo Du, Chunning Feng, Xin Fu, Chaojun Gua, Lili Hui, Dejiang Ji, Jinping Jiang, Xiaoting Li, Zhanling Liao, Feng Liu, Jingyi Liu, Shumei Ma, Fengxia Qu, Jihua Wang, Jian Xu, Xuefeng Yang, Xuecheng Yang, Wenwen Yuan, Xiaodi Zhao.

Supplementary Methods

Description of different types of health insurance schemes in China

The main health insurance schemes in China differ in their eligible population, administration, source of funding and benefits.

(i) UEBMI (Urban Employee Basic Medical Insurance), launched in 1998, is a compulsory scheme for urban employees. This scheme is funded by both employer (8-10%) and employee (2%) contributions. Retired individuals who were previously employed and covered by UEBMI remain enrolled in UEBMI.

(ii) URBMI (Urban Resident Basic Medical Insurance), launched in 2007, is a voluntary scheme for children, students, urban residents without formal employment and elderly without previous employment. This scheme is mainly funded by government subsidies (~70% of the total funds).

(iii) NRCMS (New Rural Cooperative Medical Scheme), launched in 2003, is a voluntary scheme for rural residents. This scheme is mainly funded by government subsidies (~70% of the total funds).

Imputation methods for health insurance (HI) schemes

Data on HI types by participant were identified annually in 2012-2016. Missing data on HI type for 2004-2011 were imputed based on the insurance scheme in which participants were enrolled in 2012.

Participants were linked to individual HI schemes data annually starting from 2012, using the participants' unique national ID number. Information was also provided on any uninsured participants each year.

Data for participants' HI schemes for the period 2004-2011 were imputed using the participant's earliest available HI scheme information for the period 2012 to 2016, assuming that middle-aged and older individuals in China such as participants in CKB were unlikely to change employment and, hence, their HI scheme. In China, individuals are enrolled in a particular HI scheme depending on their employment status. The proportion of CKB participants insured in each scheme remained stable between 2012 and 2016. Furthermore, at entry into the study (2004-2008), 82% of participants self-reported being insured. This proportion increased to 97% and 98% in the 5% sample of individuals who participated in the 1st resurvey (2008) and the 2nd resurvey (2013-2014), respectively. Thus, it was unlikely for participants to be uninsured.

In the analyses, URBMI and NRCMS were combined, as they provided similar benefits, and in four of the ten CKB regions, the two schemes merged into a single scheme from 2012-2013

onwards. Uninsured participants were excluded from analyses investigating effect modification by HI type, due to small number of cases.

UEBMI provides the most comprehensive coverage, while the coverage benefits of URBMI and NRCMS are similar. The cost-sharing mechanisms for each scheme vary by area, inpatient or outpatient care and by hospital tier with lower tier hospitals often associated with higher reimbursement rates and lower deductibles by insurer. Patients pay co-payments and deductibles to hospitals at the point of service, there are annual reimbursement ceilings and no ceilings on out-of-pocket spending.

Description of hospital care system in China

The Chinese hospital care system consists mainly of three levels of hospitals:

- Tier 1 or primary hospitals or health institutions: provide preventive, clinical treatment, health care and rehabilitation service in a community. Generally, a tier 1 hospital has 20–99 ward beds/
- Tier 2 or secondary hospitals: provide comprehensive medical and health services to multiple communities and offer medical training and research. Tier 2 hospitals generally have between 100 and 499 beds.
- Tier 3 or tertiary hospitals: provide high-level and specialized medical services and are responsible for higher education and scientific research. Tier 3 hospitals have at least 500 beds.

The number of doctors and nurses per bed and the medical personal's skill levels are higher in higher tier hospitals, and facilities' equipment and physical conditions tends to be of higher quality in higher tier hospitals.

Further information on data sources used

Official local residential records were used to identify about 1.8 million potentially eligible individuals, and 28% of those invited responded and enrolled in the study.

Baseline Questionnaire

Data on demographic, socioeconomic characteristics, and lifestyle and medical history were collected using an interviewer-administered, laptop-based electronic questionnaire (with logic checks to minimise missing values, errors and inconsistencies) administered to study participants. Extensive training was provided for field survey staff, including instruction on data collection using the electronic questionnaire, recording physical measurements using standard protocols and use and maintenance of equipment. Importantly, serial resurveys in 5% of the study population indicated a high level of agreement between corresponding measurements for questionnaire data and clinical measurements.

Data retrieved from medical records

Data on cardiac enzyme test, coronary angiography, electrocardiogram, echocardiogram, coronary computed tomography angiography, and Holter monitor test, were extracted from hospital medical records. Medical records were only retrieved for incident events with a main diagnosis of IHD, which were identified using regional IHD disease registries and health insurance records. Specially trained public health staff sought access to medical records of reported IHD cases in participating hospitals. A Portable Validation Device (PVD) system was

used to photograph the relevant medical records and validate the diagnosis of IHD types during follow-up. Details for each case, including dates of admission and discharge, were checked. Selected clinical data were recorded electronically using standardised, disease-specific protocols. Local regional centres' staff checked the quality and completeness of all records. If data quality was poor in a particular study region, local validation procedures were reviewed, and the retrieved data were checked. A random sample of 5% of all verified events was rechecked each year.
The most common reason for non-retrieval of medical notes was that records could not be found in the hospital. This is likely to be due to the lack of storage for medical records especially in lower tier hospitals and in rural areas.

Data sources used for analyses
When the outcomes of interest were cardiac enzyme test, coronary angiography, ECG, echocardiogram, CCTA, or Holter monitor test, the study population was only participants with retrieved medical records for first IHD admissions. Data on any use of these diagnostic tests and procedures came from the medical records.
When the outcome of interest was coronary revascularisation, the study population included all participants with a first IHD admission, regardless of whether admission's medical records were successfully retrieved. 50% of IHD admissions (AMI: 67%, angina: 82%, other IHD: 34%) had data both in HI and retrieved medical records. 45% of IHD admissions (AMI: 23%, angina: 14%, other IHD: 60%) had data in HI records only. 5% of IHD admissions (AMI: 10%, angina: 4%, other IHD: 5%), had data in retrieved medical records only.

Imputation methods for missing length of stay

For participants with retrieved admission medical records (outcomes of interest: cardiac enzyme test, coronary angiography, ECG, echocardiogram, CCTA, or Holter monitor test), 9% of IHD admissions had missing information on the length of stay (11% of admissions for men and 9% of admissions for women). For AMI, 14% of admissions had missing length of stay and the proportion was slightly lower in men (13%) than women (15%). For angina, 5% of admissions had missing length of stay and the proportion was slightly higher in men (6%) than women (4%). For other IHD, 12% of admissions had missing length of stay and the proportion was slightly higher in men (14%) than women (11%).

For all participants, regardless of whether admission medical records were retrieved (outcome of interest: coronary revascularisation), 6% of IHD admissions had missing information on the length of stay (7% of admissions for men vs 5% of admissions for women). For AMI, 11% of admissions had missing length of stay and the proportion was similar in men and women. For angina, 5% of admissions has missing length of stay and the proportion was slightly higher in men (5%) than women (4%). For other IHD, 5% of admissions had missing length of stay and the proportion was slightly higher in men (6%) than women (5%).

Checking the patterns of missingness, the odds of missing length of stay were related to several of the risk factors included in our main models. Thus, there was evidence that missing length of stay was not missing completely at random (MCAR), and we assumed that it was missing at random (MAR) and included all important covariates in its imputation. We used multiple imputation with Poisson regression and included the same covariates (eg, demographic factors, lifestyle factors, morbidity factors, health insurance type, socioeconomic factors, hospital tier and IHD type) as included in the fully adjusted models for the use of diagnostic tests and

Levy M, *et al. Heart* 2022; 108:292–299. doi: 10.1136/heartjnl-2021-318988

procedures. The number of imputations (*m*) was chosen so that m≥100*Fraction of Missing Information (White et al., 2011). Missing values were imputed using chained equations. The mean length of stay was estimated using the 10 separate datasets including imputed data with estimates combined using Rubin's rules (1987). Hence, estimates were adjusted for the variability between imputations.

Day case admissions (ie, participant admitted and discharged on the same day) were counted as having a length of stay of 0.5 days.

Description of established risk factors

Disease risk factors were classified as either demographic, lifestyle or morbidity factors. Demographic factors included annually updated age (ie, age, age-squared, age-cubed), region and calendar year. Lifestyle factors, collected at entry into the study, included smoking, alcohol consumption, body mass index, and physical activity assessed using metabolic equivalents of task (MET). Morbidity factors included systolic blood pressure (SBP), self-rated health status, self-reported mental illness, self-reported doctor-diagnosed diseases at entry into CKB, with history of major diseases updated during follow-up until censored by the onset of IHD in the present analyses.

Description of self-reported mental illness

The self-reported mental illness variable (binary) was used as a broad indicator of participants' mental health. Mental illness was defined as having at least one symptom of depression or anxiety in the past 12 months.
Symptoms of depression included feeling much more sad or depressed than usual, loss of interest in most things like hobbies or activities that usually give pleasure, loss of appetite for favourite food, or feeling worthless or useless for a period of 2 or more weeks.
Symptoms of anxiety included experiencing continuous anxiety for a period lasting one month or longer, feeling, continuous pain in body lasting more than 4 months, panic attacks, or phobias.

Description of other IHD
Admissions for other IHD [I22-I25] included a majority of atherosclerotic heart disease [I25.1].

Statistical analysis

Poisson regression was preferred to logistic regression to estimate relative risks and enable their comparison across different categories of patients, rather than odds ratios which can be misinterpreted. The use of relative risks facilitates comparisons of effect estimates across patient phenotypes with rare (i.e. use of invasive diagnostic procedures for other IHD) and frequent outcomes (i.e. use of invasive diagnostic procedures for AMI) which are required to address the hypothesis in the present study.

Our investigation strategy included all available data in the study on potential confounders or effect modifiers as informed by the literature on determinants of healthcare use. We retained all factors and did not follow data-driven model selection/building. Instead, we sequentially adjusted Poisson regression models for groups of potential confounders/effect modifiers of the

Levy M, *et al. Heart* 2022; 108:292–299. doi: 10.1136/heartjnl-2021-318988

association between gender and the use of invasive diagnostic procedures. The order in which groups of factors were included was chosen to reflect likely strength of effect and attempt to tease out the added role of socioeconomic factors. Our objective was to assess the contribution of different groups of factors that might explain the association between gender and use of procedures.

We also evaluated gender differences on an absolute scale as rates per 100 admissions of having a diagnostic test or procedure, standardised for characteristics of CKB participants with AMI, angina or other IHD in 2004-2016. Rates for urban and rural areas were standardised separately for the CKB population characteristics for the relevant urban or rural area.

Additional sensitivity analyses estimated women-to-men RRs by categories of established IHD risk factors except for smoking and alcohol consumption because of their low prevalence in Chinese women.

Levy M, *et al. Heart* 2022; 108:292–299. doi: 10.1136/heartjnl-2021-318988

eTable 1: Selected characteristics of men and women with retrieved medical records for hospital admissions for acute MI, angina and other IHD, in 2004-2016

	**AMI**			**Angina**			**Other IHD**		
	**Men (n=1558)**	**Women (n=937)**		**Men (n=3549)**	**Women (n=6284)**		**Men (n=4483)**	**Women (n=7597)**	
**(A) Characteristics at baseline**									
Age (years), Mean (SD)	58.8 (10.1)	62.4 (8.8)	**	58.3 (10.1)	58.7 (9.2)		60.9 (9.6)	59.2 (9.5)	**
**Prior medical history, %**									
Diabetes[1]	14.4	26.5	**	14.2	15		10.6	12.5	**
Hypertension[1]	55.7	60.7	*	52.5	44.7	**	55.1	48.9	*
Stroke or TIA	7.8	3.6	**	5.9	4.1	**	5	3.3	**
IHD	11.7	16.1	**	18.7	22	**	14.5	14.7	
CKD	1.7	2.1		3.2	3.9		1.6	2.3	**
Poor health status	11.5	19.1	**	12.7	16.2	**	13.8	18.7	
Mental illness[2]	9.9	11.4		9.7	13.5	**	10.6	12.9	
**Physical measurements**									
Overweight or obese (>25 kg/m²), %	44.8	48.8		51.6	52.4		38.9	48.2	
SBP (mmHg), Mean (SD)	142.3 (22.1)	145.8 (24.4)	**	138.8 (20.8)	134,8 (23.2)	**	140.5 (21.9)	138.3 (23.9)	**
**Lifestyle characteristics**									
Current smoker, %	62.6	6.7	**	51.6	3.9	**	53.2	3.9	**
Regular alcohol drinker, %	41.5	3.3	**	47.9	5.3	**	40.2	4	**
Physical activity (MET-h/day), Mean (SD)	15.6 (13.7)	12.7 (8.5)	**	14.2 (11.5)	13.2 (8.3)	**	15.4 (13.3)	14.1 (9.3)	**
**Socioeconomic characteristics**									
Currently married, %	94	76.6	**	94.3	82.4	**	90.7	82	**
Household size, Mean (SD)	3.5 (1.6)	3.3 (1.6)	**	3.2 (1.4)	3.1 (1.4)	**	3.6 (1.7)	3.5 (1.7)	*
High School or above, %	28.4	13.4	**	43.6	32.2	**	26.3	18.9	**
Annual household income >20,000¥, %	46.6	31.2	**	53.8	46.1	**	43.3	36.4	**
Rural residents, %	34.8	37.7		21	14.2	**	51.2	47.4	**
**(B) Characteristics at hospital admission**									
**Age (years), Mean (SD)**	64.9 (9.9)	68.6 (8.8)	**	64.5 (9.9)	64.9 (9.0)	*	67.3 (9.5)	65.8 (9.4)	**
**Health insurance type[3], %**			**						**
NRCMS or URBMI	36.7	48.6		19.6	19.5		48.8	50.5	
UEBMI	61.9	50.4		79.6	80.1		50.2	49	
Other or uninsured	1.4	1.1		0.7	0.5		1	0.5	
**Hospital tier, %**			*						
Tier 1 or missing/unspecified	9.2	12.3		10	10.2		29.4	32.7	
Tier 2	14.6	16.8		16.8	17.3		21.5	19.9	*
Tier 3	76.3	71		73.2	72.6		49.1	47.4	
**Length of stay (days)[4], Mean (SD)**	10.5 (5.9)	10.7 (5.8)		10.4 (5.6)	9.1 (5.2)		10.8 (5.3)	9.4 (5.5)	

MI: myocardial infarction, IHD: ischaemic heart disease, CKD: chronic kidney disease, SD: standard deviation, TIA: transient ischemic attack, MET: metabolic equivalents of task, URBMI: Urban Resident Basic Medical Insurance, NRCMS: New Rural Cooperative Medical Scheme, UEBMI: Urban Employee Basic Medical Insurance. ** p-value <0.01 and * p-value < 0.5. [1]Self-reported and screen-detected. [2]Mental illness was defined as having at least one symptom of depression or anxiety in the past 12 months. [3]Data on health insurance (HI) types for each participant was identified annually in 2012-2016, but was unavailable for the years prior to 2012. Missing data on HI type in 2004-2011 were imputed based on the insurance scheme in which participants were enrolled in 2012. [4]Missing length of stay (14% for AMI and 9% for other IHD) was imputed using multiple imputation.

Levy M, *et al. Heart* 2022; 108:292–299. doi: 10.1136/heartjnl-2021-318988

eTable 2: Adjusted rates (95% CI) of having cardiac enzyme test, coronary angiography and coronary revascularisation for ACUTE MI per 100 admissions, by gender and socioeconomic and health system factors

	Cardiac enzymes test		Coronary angiography		Coronary revascularisation	
	**Men**	**Women**	**Men**	**Women**	**Men**	**Women**
**Area of residence**						
Urban	94.7 (93.1, 96.3)	94.4 (92.4, 96.5)	38.3 (34.8, 41.8)	31.3 (27.5, 35.2)	35.6 (32.6, 38.6)	31.1 (27.5, 34.7)
Rural	84.8 (81.1, 88.5)	85.2 (81.3, 89.1)	13.8 (11.4, 16.1)	9.7 (7.3, 12.1)	8.9 (7.3, 10.6)	7.1 (5.3, 8.9)
**Current marital status**						
Married	91.7 (89.8, 93.7)	91.6 (89.4, 93.8)	29.9 (27.3, 32.5)	23.1 (20.2, 26.0)	25.4 (23.3, 27.4)	21.3 (18.8, 23.9)
Other	88.0 (82.1, 93.9)	89.4 (85.5, 93.3)	26.6 (17.7, 35.5)	27.3 (21.0, 33.5)	18.9 (13.0, 24.8)	18.5 (14.8, 22.2)
**Health insurance type**						
URBMI or NRCMS	85.6 (82, 89.1)	86.9 (83.6, 90.3)	15.7 (13.2, 18.3)	12.4 (10.1, 14.7)	26.4 (21.1, 31.7)	22.7 (18.2, 27.2)
UEBMI	94.6 (93, 96.3)	93.8 (91.5, 96.0)	38.2 (34.7, 41.7)	30.5 (26.5, 34.6)	24.1 (22.0, 26.3)	20.5 (17.8, 23.1)
**Education**						
No formal school	87.4 (82.0, 92.7)	90.1 (87.0, 93.2)	15.9 (10.8, 21.0)	15.7 (12.3, 19.0)	15.4 (11.7, 19.2)	12.1 (9.7, 14.6)
Primary/Middle school	90.8 (88.6, 92.9)	90.3 (87.7, 92.9)	29.7 (26.8, 32.6)	23.0 (19.7, 26.2)	22.7 (20.5, 24.8)	19.7 (17.0, 22.3)
High school and above	93.9 (91.8, 96.0)	93.6 (89.3, 97.8)	37.2 (32.8, 41.7)	29.2 (22.2, 36.2)	34.9 (31.1, 38.7)	29.9 (23.2, 36.5)
**Annual household income (Yuan)**						
<10,000	92.1 (88.4, 95.7)	93.5 (89.8, 97.2)	29.2 (23.3, 35.1)	21.5 (16.2, 26.8)	24.6 (19.8, 29.4)	19.8 (15.1, 24.5)
10,000-19,999	92.3 (90.1, 94.5)	90.1 (87.0, 93.2)	29.3 (25.8, 32.8)	22.7 (18.6, 26.8)	23.1 (20.4, 25.8)	21.7 (18.3, 25.2)
20,000+	90.0 (87.5, 92.4)	91.3 (88.5, 94.1)	30.5 (27.2, 33.8)	25.6 (21.4, 29.8)	25.7 (23.1, 28.2)	20.8 (17.4, 24.1)
**Hospital tier**						
Tier 3	95.5 (94.0, 97.1)	95.5 (93.6, 97.3)	44.0 (40.2, 47.8)	36.7 (32.5, 40.8)	36.9 (33.7, 40.2)	32.8 (29.2, 36.4)
Other	86.7 (83.0, 90.4)	86.8 (82.3, 91.3)	11.2 (7.9, 14.6)	4.3 (1.6, 6.9)	10.1 (6.8, 13.3)	4.6 (2.0, 7.1)
**Calendar period**						
2004-2011	88.3 (85.6, 90.9)	87.8 (84.2, 91.4)	22.8 (19.9, 25.8)	18.4 (14.5, 22.2)	19.6 (17.4, 21.7)	14.9 (12, 17.8)
2012-2014	92.8 (90.5, 95.1)	92.8 (89.9, 95.6)	29.5 (26.1, 32.9)	22.7 (18.7, 26.7)	27.3 (24.3, 30.2)	25.4 (21.4, 29.3)
2015-2016	93.4 (90.5, 96.3)	93.6 (92.3, 94.9)	39.6 (34.8, 44.4)	32.5 (26.8, 38.3)	30.3 (26.6, 34.0)	26.6 (21.9, 31.2)
**Overall**	**93.6 (92.3, 94.9)**	**93.7 (91.7, 95.6)**	**34.9 (32.5, 37.2)**	**27.8 (24.5, 31.0)**	**32.3 (30.3, 34.3)**	**27.6 (24.5, 30.7)**

UEBMI: Urban Employee Basic Medical Insurance, URBMI: Urban Resident Basic Medical Insurance, NRCMS: New Rural Cooperative Medical Scheme. Models included adjustments for demographic factors, lifestyle factors, morbidity factors, health insurance type (except by area of residence), socioeconomic factors, and hospital tier, as appropriate. In analyses by HI type, uninsured participants were excluded due to small number of cases. For all factors except area of residence, rates were standardised for the overall CKB participant population with AMI or other IHD in 2004-2016. Probabilities by area of residence were standardised separately for the CKB participant population living in urban or rural area, as appropriate. Coronary revascularisations within 3 months post admission were included.

eTable 3: Adjusted rates (95% CI) of having cardiac enzyme test, coronary angiography and coronary revascularisation for ANGINA per 100 admissions, by gender and socioeconomic and health system factors

	Cardiac enzymes test		Coronary angiography		Coronary revascularisation	
	**Men**	**Women**	**Men**	**Women**	**Men**	**Women**
**Area of residence**						
Urban	77.8 (75.9, 79.6)	74.7 (73.3, 76.1)	22.5 (20.6, 24.3)	14.9 (13.6, 16.1)	14.1 (12.4, 15.7)	7.8 (6.8, 8.7)
Rural	57.8 (52.4, 63.2)	57.6 (52.3, 62.8)	8.7 (7.0, 10.3)	6.0 (4.7, 7.3)	3.8 (2.1, 5.5)	2.7 (1.6, 3.8)
**Current marital status**						
Married	71.1 (68.7, 73.4)	68.8 (66.7, 70.9)	17.9 (16.5, 19.3)	11.8 (10.8, 12.8)	10.1 (8.8, 11.4)	5.6 (4.8, 6.4)
Other	71.4 (65.9, 76.9)	67.8 (64.8, 70.7)	17.2 (12.4, 21.9)	11.1 (9.1, 13.1)	7.4 (4.2, 10.6)	5.5 (4.2, 6.9)
**Health insurance type**						
URBMI or NRCMS	58.9 (54.3, 63.4)	60 (55.8, 64.2)	10.8 (8.8, 12.9)	7.9 (6.6, 9.3)	8.8 (5.9, 11.8)	6.6 (4.8, 8.3)
UEBMI	77.6 (75.8, 79.3)	74.1 (72.7, 75.5)	21.7 (20, 23.4)	14.1 (13, 15.3)	10.1 (8.6, 11.6)	5.4 (4.6, 6.3)
**Education**						
No formal school	67.3 (58.6, 76.0)	63.6 (57.3, 70.0)	8.4 (4.0, 12.8)	8.2 (6.5, 9.8)	7.6 (3.0, 12.2)	4.2 (2.7, 5.7)
Primary/Middle school	71.4 (69.2, 73.7)	68.7 (66.8, 70.6)	17.9 (16.2, 19.6)	12.2 (11.0, 13.4)	9.6 (8.2, 11.1)	5.9 (5.0, 6.8)
High school and above	73.0 (70.8, 75.1)	71.1 (69.1, 73.1)	21.1 (19.0, 23.2)	12.8 (11.1, 14.4)	11.8 (10.2, 13.4)	5.8 (4.6, 7.0)
**Annual household income (Yuan)**						
<10,000	76.8 (72.6, 81.0)	70.2 (67.1, 73.3)	15.4 (12.2, 18.5)	12 (9.9, 14.2)	7.6 (5.2, 10.0)	6.1 (4.5, 7.8)
10,000-19,999	69.9 (67.0, 72.8)	68.4 (66.0, 70.8)	16.9 (14.9, 18.8)	12.3 (10.8, 13.7)	9.4 (7.8, 11.1)	5.9 (4.8, 7.0)
20,000+	69.8 (67.2, 72.4)	67.9 (65.6, 70.2)	19.0 (17.0, 21.0)	11.3 (10.1, 12.5)	10.7 (9.0, 12.3)	5.2 (4.3, 6.1)
**Hospital tier**						
Tier 3	82.0 (79.6, 84.3)	80.3 (78.3, 82.3)	26.4 (24.2, 28.5)	17.5 (16.1, 18.9)	17.7 (15.1, 20.3)	9.9 (8.4, 11.4)
Other	59.0 (55.5, 62.4)	53.6 (50.7, 56.4)	5.5 (4.1, 6.8)	3.3 (2.4, 4.1)	1.6 (0.9, 2.3)	1.1 (0.6, 1.5)
**Calendar period**						
2004-2011	61.5 (58.7, 64.4)	55.7 (53.4, 58.1)	15.2 (13.3, 17.0)	10.8 (9.4, 12.1)	8.7 (7.2, 10.3)	5.1 (4.1, 6.1)
2012-2014	73.7 (71.0, 76.5)	73.1 (70.7, 75.5)	18.9 (16.9, 21.0)	12.2 (10.8, 13.5)	11.2 (9.4, 13.1)	6.4 (5.3, 7.6)
2015-2016	78.3 (75.2, 81.3)	79.0 (76.3, 81.6)	19.9 (17.5, 22.3)	12.7 (11.1, 14.4)	10.1 (8.2, 12.0)	5.3 (4.2, 6.5)
**Overall**	**75.1 (73.4, 76.7)**	**72.5 (71.4, 73.6)**	**18.3 (16.9, 19.7)**	**12.1 (11.2, 13)**	**10.8 (9.6, 12)**	**6.1 (5.4, 6.7)**

UEBMI: Urban Employee Basic Medical Insurance, URBMI: Urban Resident Basic Medical Insurance, NRCMS: New Rural Cooperative Medical Scheme. Models included adjustments for demographic factors, lifestyle factors, morbidity factors, health insurance type (except by area of residence), socioeconomic factors, and hospital tier, as appropriate. In analyses by HI type, uninsured participants were excluded due to small number of cases. For all factors except area of residence, rates were standardised for the overall CKB participant population with AMI or other IHD in 2004-2016. Probabilities by area of residence were standardised separately for the CKB participant population living in urban or rural area, as appropriate. Coronary revascularisations within 3 months post admission were included.

eTable 4: Adjusted rates (95% CI) of having cardiac enzymes test, coronary angiography or coronary revascularisation for OTHER IHD per 100 admissions for men and women, by socioeconomic and health system factors

	Cardiac enzymes test		Coronary angiography		Coronary revascularisation	
	**Men**	**Women**	**Men**	**Women**	**Men**	**Women**
**Area of residence**						
Urban	69.2 (67.2, 71.2)	67.5 (66.1, 69)	13.2 (11.9, 14.4)	11.5 (10.5, 12.4)	5.1 (4.5, 5.8)	3.1 (2.7, 3.5)
Rural	59.7 (57.7, 61.7)	56.8 (55.2, 58.4)	4.1 (3.3, 5.0)	3.3 (2.6, 3.9)	2.5 (2.1, 3.0)	1.5 (1.2, 1.8)
**Current marital status**						
Married	65.7 (64.1, 67.3)	63.8 (62.5, 65.1)	10.2 (9.3, 11.2)	8.8 (8.0, 9.5)	4.3 (3.8, 4.8)	2.5 (2.2, 2.8)
Other	67.7 (63.7, 71.7)	64.4 (62.0, 66.7)	8.0 (5.4, 10.6)	7.6 (6.2, 9.1)	1.8 (0.8, 2.7)	2.1 (1.6, 2.7)
**Health insurance type**						
URBMI or NRCMS	61.3 (59.2, 63.4)	58.7 (57.1, 60.2)	5.6 (4.7, 6.5)	4.8 (4.1, 5.6)	3.5 (2.8, 4.1)	2.3 (1.8, 2.8)
UEBMI	68.4 (66.5, 70.4)	66.9 (65.4, 68.4)	12.8 (11.5, 14)	10.8 (9.8, 11.7)	4.3 (3.8, 4.9)	2.5 (2.1, 2.9)
**Education**						
No formal school	69.2 (64.6, 73.9)	64.6 (62.7, 66.6)	5.9 (4.1, 7.8)	5.0 (4.2, 5.9)	2.7 (1.8, 3.7)	2.6 (2.1, 3.1)
Primary/Middle school	65.5 (63.6, 67.3)	64.2 (62.8, 65.7)	10.2 (9.1, 11.3)	9.0 (8.1, 9.9)	4.4 (3.8, 4.9)	2.5 (2.2, 2.9)
High school and above	65.7 (63.1, 68.2)	62.8 (60.4, 65.1)	11.9 (10.3, 13.5)	10.1 (8.8, 11.5)	3.8 (3.1, 4.5)	2.0 (1.5, 2.6)
**Annual household income (Yuan)**						
<10,000	67.6 (64.4, 70.8)	66.2 (63.6, 68.7)	9.2 (7.1, 11.4)	7.4 (5.8, 8.9)	3.4 (2.4, 4.3)	2.2 (1.6, 2.8)
10,000-19,999	65.7 (63.2, 68.2)	64.2 (62.3, 66.1)	9.7 (8.3, 11.2)	9.1 (7.9, 10.2)	4.1 (3.4, 4.8)	2.6 (2.1, 3.1)
20,000+	65.3 (63.2, 67.4)	62.4 (60.7, 64.1)	10.4 (9.2, 11.5)	8.8 (7.9, 9.7)	4.2 (3.6, 4.8)	2.5 (2.1, 2.9)
**Hospital tier**						
Tier 3	73.0 (71.1, 75.0)	74.6 (73.0, 76.1)	15.3 (13.9, 16.7)	13.3 (12.2, 14.4)	7.4 (6.4, 8.4)	4.5 (3.9, 5.1)
Other	52.9 (50.9, 54.8)	47.1 (45.6, 48.5)	1.6 (1.1, 2.1)	1.3 (1.0, 1.7)	0.6 (0.4, 0.9)	0.3 (0.2, 0.5)
**Calendar period**						
2004-2011	53.3 (50.9, 55.8)	50.4 (48.4, 52.5)	6.9 (5.8, 8.0)	5.6 (4.7, 6.5)	2.9 (2.4, 3.4)	1.8 (1.4, 2.1)
2012-2014	70.5 (68.3, 72.8)	69.5 (67.8, 71.2)	9.7 (8.4, 10.9)	9.0 (8.0, 10.1)	4.3 (3.6, 5.1)	2.4 (1.9, 2.9)
2015-2016	73.6 (71.0, 76.1)	72.5 (70.5, 74.4)	14.3 (12.4, 16.1)	12.5 (11.1, 14).0	5.0 (4.2, 5.9)	3.6 (2.9, 4.2)
**Overall**	**64.6 (63.1, 66.1)**	**62.5 (61.4, 63.7)**	**9.9 (9.1, 10.8)**	**8.6 (7.9, 9.3)**	**3.7 (3.3, 4.1)**	**2.3 (2.0, 2.5)**

UEBMI: Urban Employee Basic Medical Insurance, URBMI: Urban Resident Basic Medical Insurance, NRCMS: New Rural Cooperative Medical Scheme. Models included adjustments for demographic factors, lifestyle factors, morbidity factors, health insurance type (except by area of residence), socioeconomic factors, and hospital tier, as appropriate. In analyses by HI type, uninsured participants were excluded due to small number of cases. For all factors except area of residence, rates were standardised for the overall CKB participant population with AMI or other IHD in 2004-2016. Probabilities by area of residence were standardised separately for the CKB participant population living in urban or rural area, as appropriate. Coronary revascularisations within 3 months post admission were included.

eTable 5: Adjusted rates (95% CI) of having other diagnostic tests per 100 admissions for acute MI, angina and other IHD, by gender

	Acute MI		Angina		Other IHD	
	**Men**	**Women**	**Men**	**Women**	**Men**	**Women**
**ECG**	98.5 (97.6, 99.5)	98.4 (97.5, 99.3)	98.1 (97.5, 98.7)	97.9 (97.4, 98.4)	96.1 (95.4, 96.9)	96.5 (96.0, 97.0)
**Echocardiogram**	63.2 (60.3, 66.1)	60.4 (57.3, 63.6)	67.4 (65.8, 69.0)	65.8 (64.6, 67.1)	63.5 (61.8, 65.2)	63.1 (61.9, 64.3)
**Holter monitor test**	24.0 (21.2, 26.9)	24.9 (21.7, 28.0)	35.8 (33.1, 38.5)	37.5 (34.9, 40.1)	33.9 (32.1, 35.6)	33.8 (32.6, 35.0)
**CCTA**	8.2 (6.5, 10.0)	8.5 (6.5, 10.6)	11.0 (9.8, 12.2)	9.9 (9.1, 10.8)	10.8 (9.7, 12.0)	9.8 (9.0, 10.6)

MI: myocardial infarction, IHD: ischaemic heart disease, ECG: electrocardiogram, CCTA: coronary computed tomography angiography. Models included adjustments for demographic factors, lifestyle factors, morbidity factors, health insurance type, socioeconomic factors, and hospital tier, as appropriate. Rates were standardised for the overall CKB participant population with AMI or other IHD in 2004-2016.

eTable 6: Adjusted women-to-men rate ratios and rates of 28-days case fatality for acute MI and other IHD for men and women

	Rates per 100 admissions (95% CI)				Women-to-men rate ratios (95% CI)	
	Acute MI		Other IHD		Acute MI	Other IHD
	**Men (n=165)**	**Women (n=123)**	**Men (n=166)**	**Women (n=147)**		
**Unadjusted**	9.2 (7.8, 10.6)	11.1 (9.1, 13.0)	1.7 (1.5, 2)	0.9 (0.7, 1)	1.21 (0.96, 1.52)	0.51 (0.41, 0.64)
**+ age, region, year**	9.8 (8.3, 11.3)	10.0 (8.2, 11.7)	1.6 (1.4, 1.9)	0.9 (0.8, 1.1)	1.01 (0.80, 1.28)	0.58 (0.46, 0.72)
**+ lifestyle and morbidity factors**	10.7 (8.9, 10.5)	9.0 (7.2, 10.7)	1.5 (1.3, 1.8)	1.0 (0.8, 1.1)	0.84 (0.63, 1.11)	0.61 (0.46, 0.81)

MI: myocardial infarction, IHD: ischaemic heart disease. Data used was from HI records supplemented by retrieved medical records. Results using data from retrieved medical records only were slightly higher. Case fatality rates for angina were not presented due to small number of 28-day case fatalities (men: 21, women: 19).

Levy M, *et al. Heart* 2022; 108:292–299. doi: 10.1136/heartjnl-2021-318988

eFigure 1: Adjusted women-to-men rate ratios of having coronary angiography for acute MI, angina and other IHD, by socioeconomic and health system factors

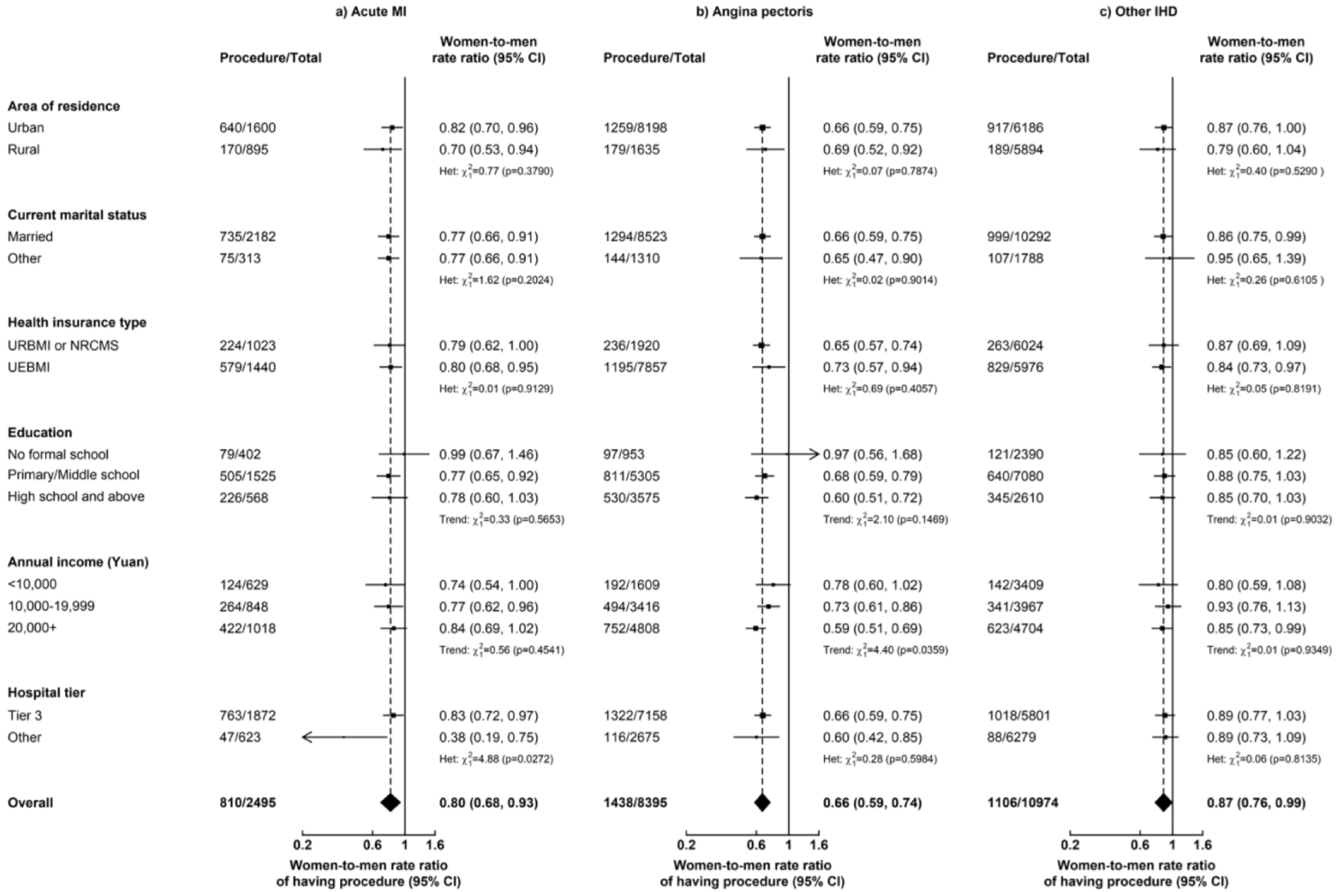

MI: myocardial infarction, IHD: ischaemic heart disease. UEBMI: Urban Employee Basic Medical Insurance, URBMI: Urban Resident Basic Medical Insurance, NRCMS: New Rural Cooperative Medical Scheme. In analyses by health insurance type, uninsured participants were excluded due to small number of cases. Models included adjustments for demographic factors, lifestyle factors, morbidity factors, health insurance type (except by area of residence), socioeconomic factors, and hospital tier, as appropriate. The area of each square is inversely proportional to the variance

eFigure 2: Adjusted women-to-men rate ratios of having cardiac enzyme tests, coronary angiography and coronary revascularisation for STEMI and NSTEMI, after sequential adjustment for confounding factors

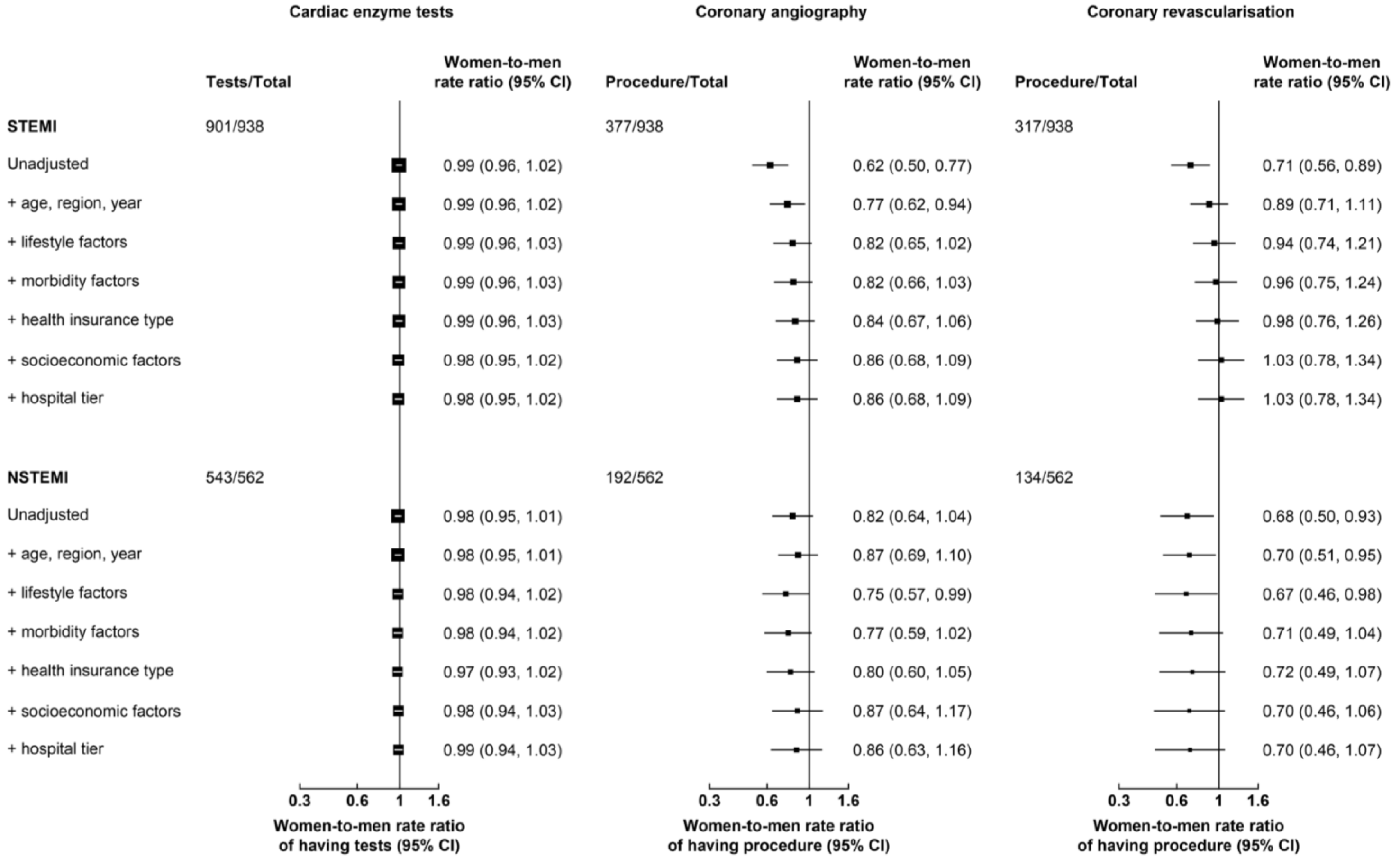

STEMI: ST segment elevation myocardial infarction myocardial infarction, NSTEMI: non-ST segment elevation myocardial infarction. Data on AMI subtypes were only available in 1500 AMI admissions with retrieved medical records. The area of each square is inversely proportional to the variance.

Levy M, *et al. Heart* 2022; 108:292–299. doi: 10.1136/heartjnl-2021-318988

eFigure 3: Adjusted women-to-men rate ratios of having cardiac enzyme test, coronary angiography and coronary revascularisation for FIRST-EVER ACUTE MI, angina and other IHD, by level of adjustment

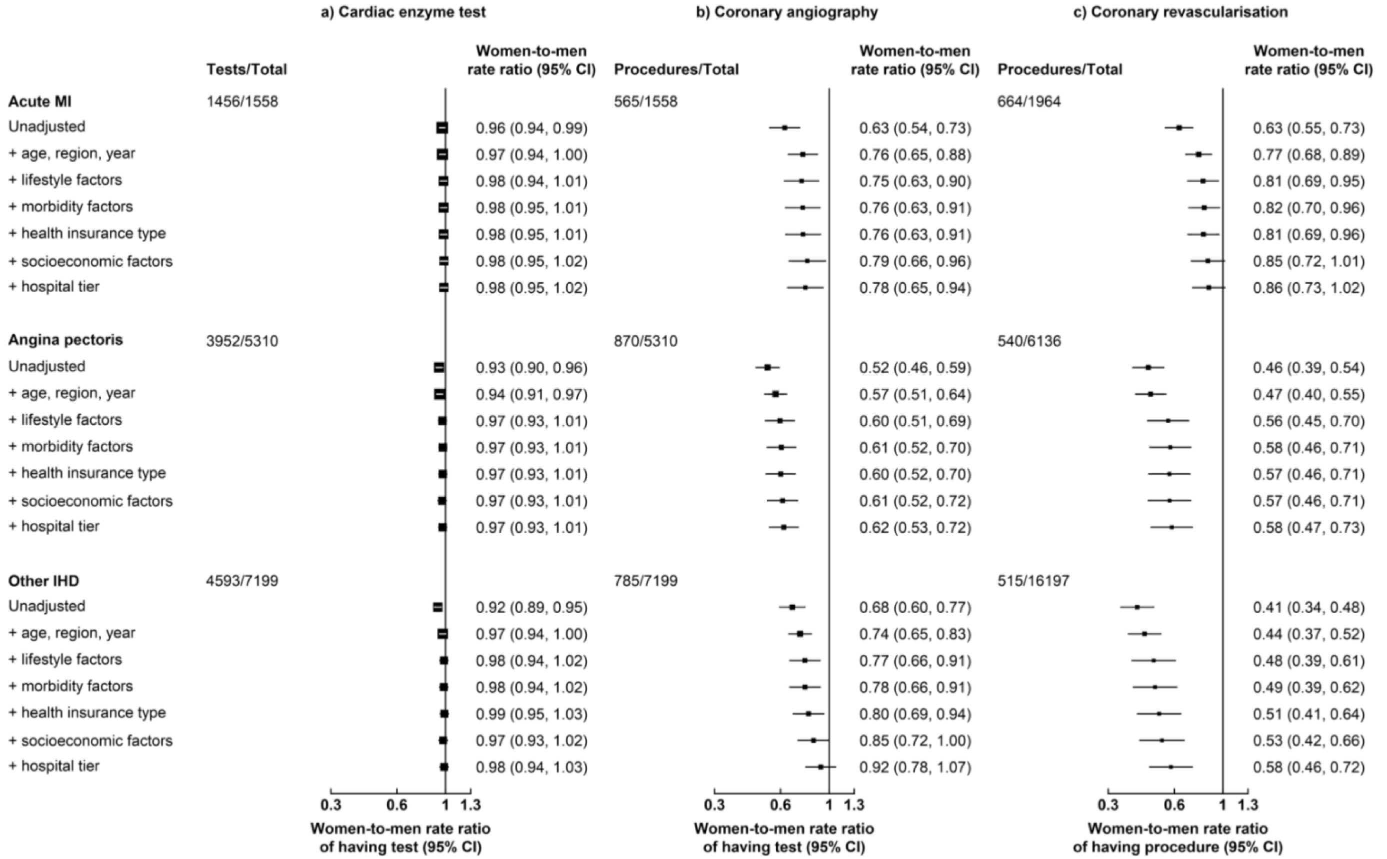

MI: myocardial infarction, IHD: ischaemic heart disease. Coronary revascularisations within 3 months post admission were included. The total number for analyses of cardiac enzyme test and coronary angiography included first-ever admissions for participants with retrieved medical records. The total number for analyses of coronary revascularisation included all first-ever IHD admissions for participants with and without retrieved medical records. The area of each square is inversely proportional to the variance.

Levy M, *et al. Heart* 2022; 108:292–299. doi: 10.1136/heartjnl-2021-318988

eFigure 4: Adjusted women-to-men rate ratios of having cardiac enzyme test, coronary angiography and coronary revascularisation for acute MI, angina and other IHD by level of adjustment, analyses stratified by region

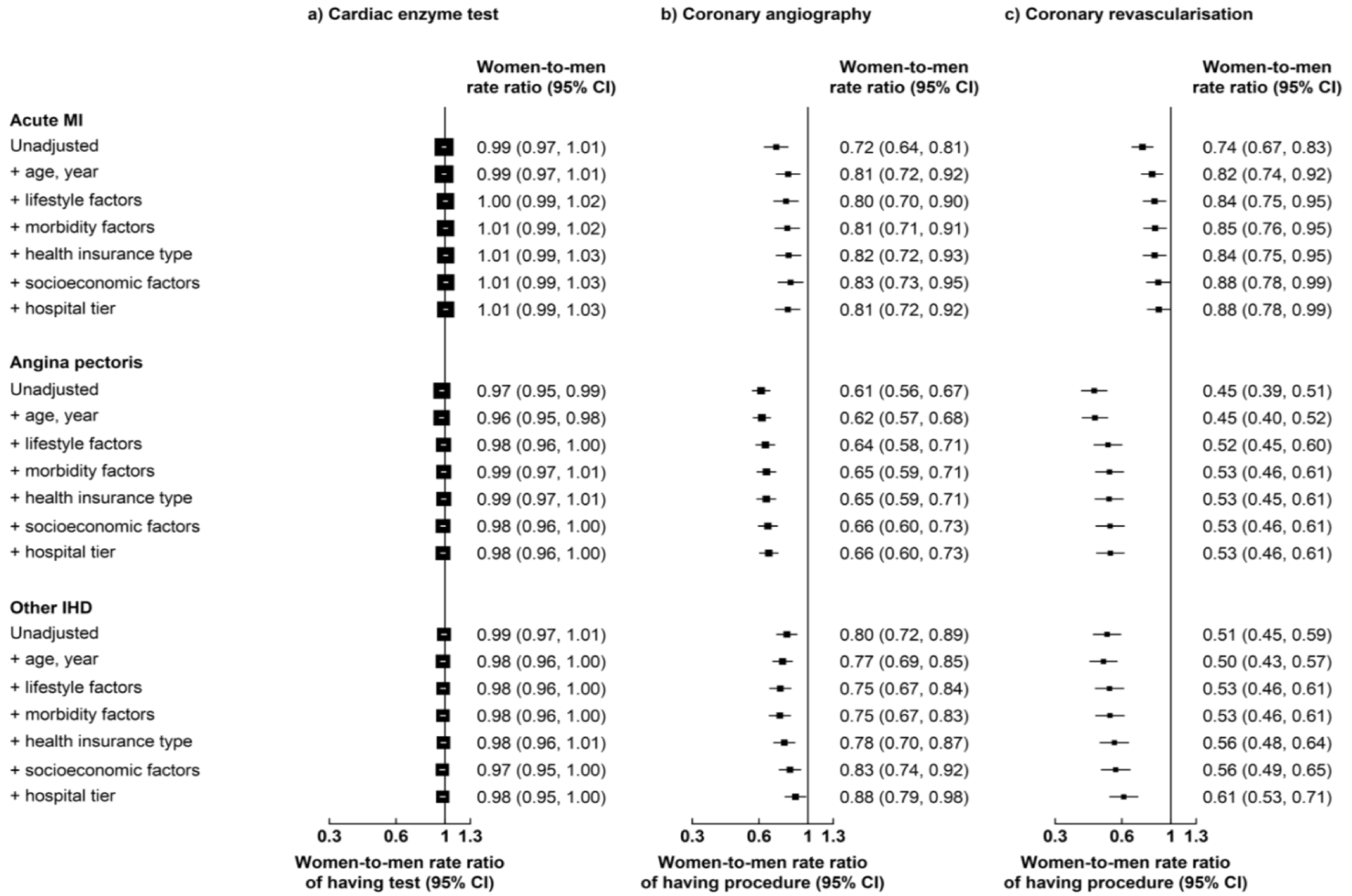

MI: myocardial infarction, IHD: ischaemic heart disease Coronary revascularisations within 3 months post admission were included. The area of each square is inversely proportional to the variance. Estimates were synthesized using inverse-variance weighting method.

eFigure 5: Adjusted women-to-men rate ratios of having cardiac enzyme test, coronary angiography and coronary revascularisation for ACUTE MI, by selected risk factors

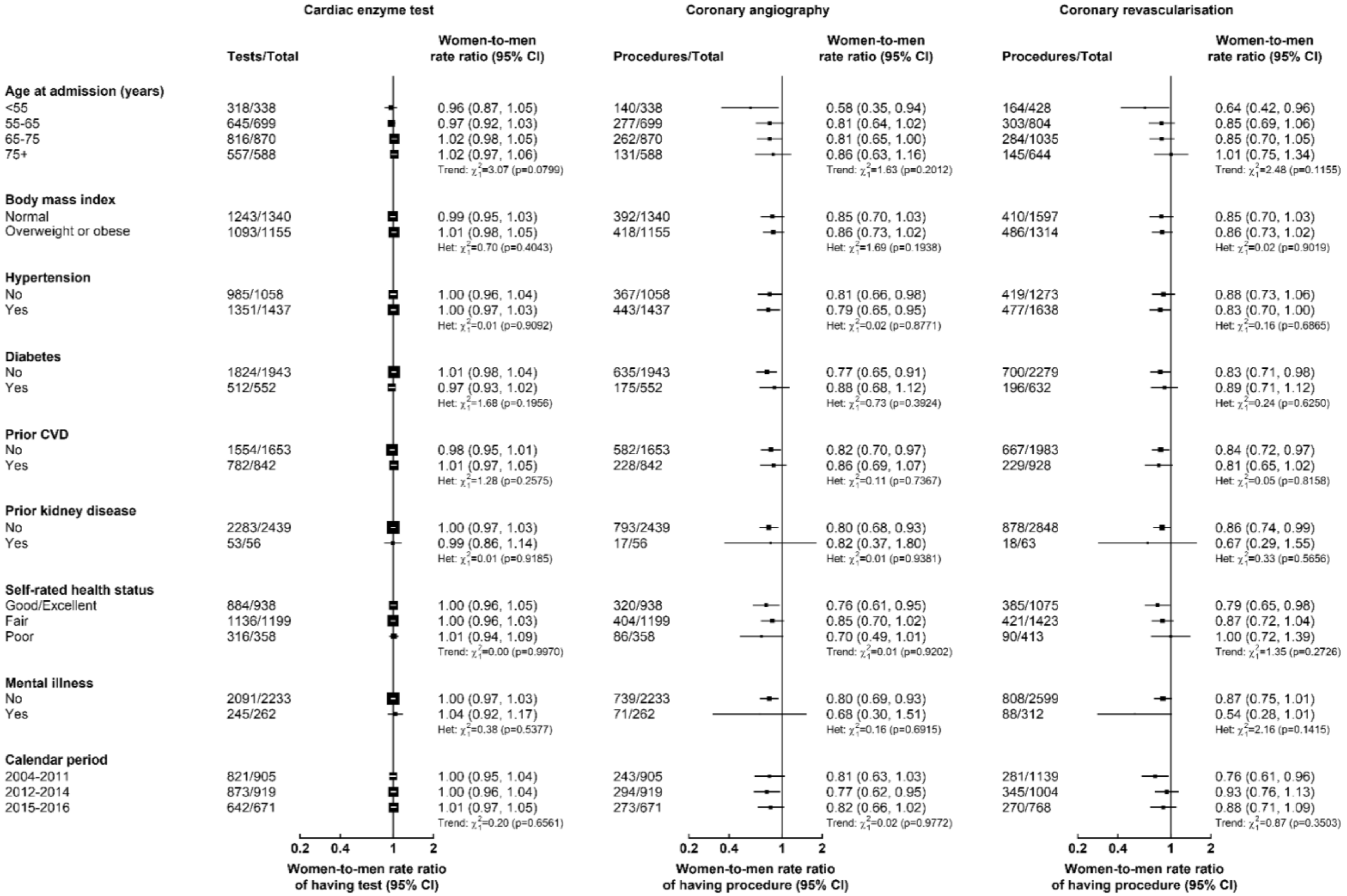

MI: myocardial infarction, CVD: cardiovascular disease. Models included adjustments for demographic factors, lifestyle factors, morbidity factors, health insurance type (except by area of residence), socioeconomic factors, and hospital tier, as appropriate. Coronary revascularisations within 3 months post admission were included. The area of each square is inversely proportional to the variance.

Levy M, *et al. Heart* 2022; 108:292–299. doi: 10.1136/heartjnl-2021-318988

eFigure 6: Adjusted women-to-men rate ratios of having cardiac enzyme test, coronary angiography and coronary revascularisation for ANGINA, by selected risk factors

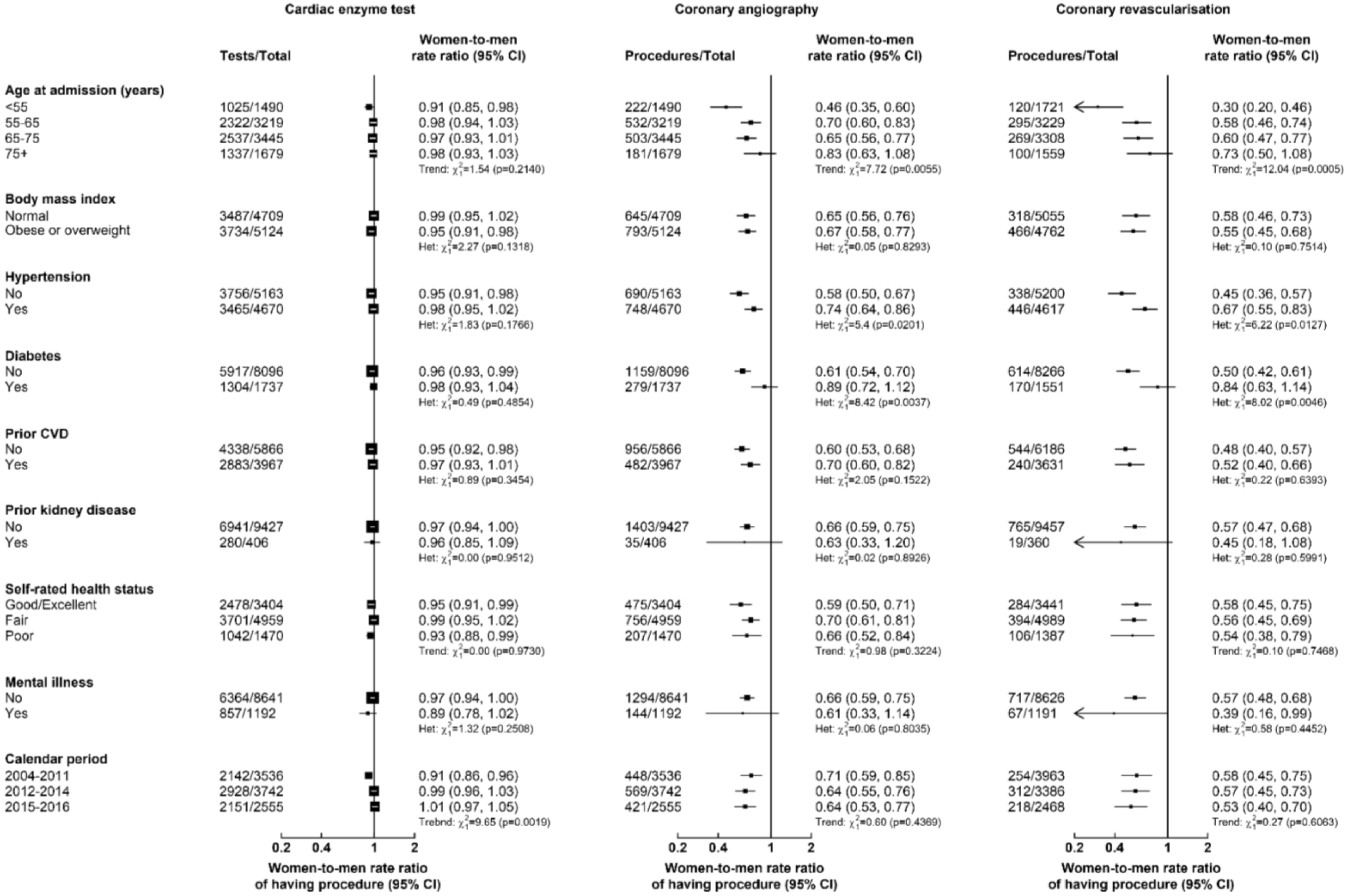

CVD: cardiovascular disease. Models included adjustments for demographic factors, lifestyle factors, morbidity factors, health insurance type (except by area of residence), socioeconomic factors, and hospital tier, as appropriate. Coronary revascularisations within 3 months post admission were included. The area of each square is inversely proportional to the variance.

Levy M, *et al. Heart* 2022; 108:292–299. doi: 10.1136/heartjnl-2021-318988

eFigure 7: Adjusted women-to-men rate ratios of having cardiac enzyme test, coronary angiography and coronary revascularisation for OTHER IHD, by selected risk factors

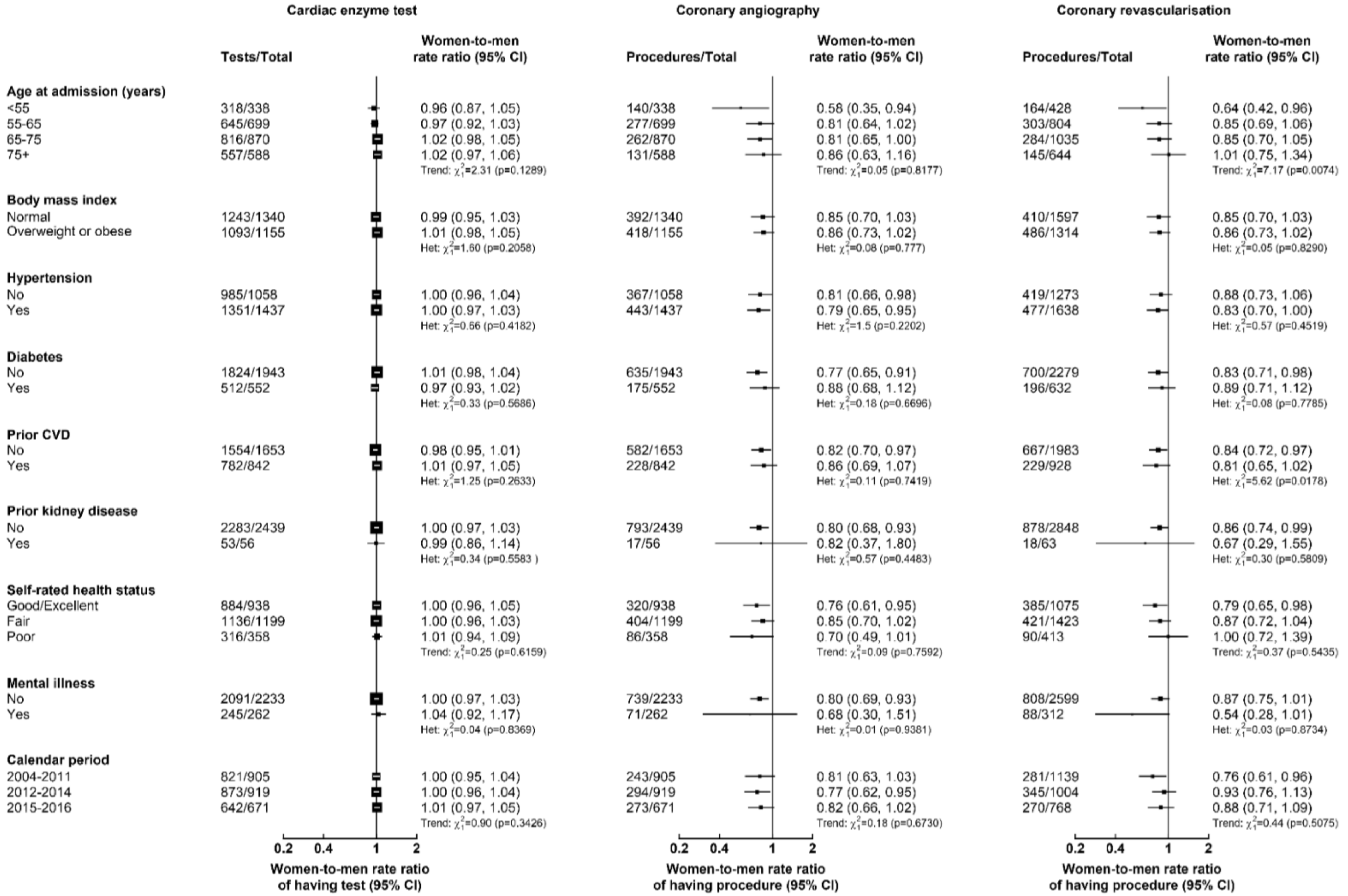

IHD: ischaemic heart disease. CVD: cardiovascular disease. Models included adjustments for demographic factors, lifestyle factors, morbidity factors, health insurance type (except by area of residence), socioeconomic factors, and hospital tier, as appropriate. Coronary revascularisations within 3 months post admission were included. The area of each square is inversely proportional to the variance.

Levy M, *et al. Heart* 2022; 108:292–299. doi: 10.1136/heartjnl-2021-318988
